# Iron limitation indirectly reduces the *Escherichia coli torCAD* operon expression by a reduction of molybdenum cofactor availability

**DOI:** 10.1128/spectrum.03480-23

**Published:** 2024-01-09

**Authors:** Muhammad Abrar Hasnat, Arkadiusz Zupok, Michal Gorka, Chantal Iobbi-Nivol, Aleksandra Skirycz, Cécile Jourlin-Castelli, Frank Bier, Saloni Agarwal, Ehizode Irefo, Silke Leimkühler

**Affiliations:** 1 Department of Molecular Enzymology, Institute of Biochemistry and Biology, University of Potsdam, Potsdam, Germany; 2 Max-Planck-Institute of Molecular Plant Physiology, Potsdam, Germany; 3 Laboratoire de Bioénergétique et Ingénierie des Protéines, Institut de Microbiologie de la Méditerranée, Centre National de la Recherche Scientifique, Aix-Marseille Université, Marseille, France; 4 Department of Molecular Bioanalytics and Bioelectronics, Institute of Biochemistry and Biology, University of Potsdam, Potsdam, Germany; Forschungszentrum Jülich GmbH, Juelich, Germany

**Keywords:** iron regulation, *Escherichia coli*, molybdenum cofactor, FNR, iron-sulfur cluster, anaerobic respiration, TMAO reductase, *torCAD*, IscR

## Abstract

The expression of most molybdoenzymes in *Escherichia coli* has so far been revealed to be regulated by anaerobiosis and requires the presence of iron, based on the necessity of the transcription factor FNR to bind one [4Fe-4S] cluster. One exception is trimethylamine-*N*-oxide reductase encoded by the *torCAD* operon, which has been described to be expressed independently from FNR. In contrast to other alternative anaerobic respiratory systems, the expression of the *torCAD* operon was shown not to be completely repressed by the presence of dioxygen. To date, the basis for the O_2_-dependent expression of the *torCAD* operon has been related to the abundance of the transcriptional regulator IscR, which represses the transcription of *torS* and *torT,* and is more abundant under aerobic conditions than under anaerobic conditions. In this study, we reinvestigated the regulation of the *torCAD* operon and its dependence on the presence of iron and identified a novel regulation that depends on the presence of the bis-molybdopterin guanine dinucleotide (bis-MGD) molybdenum cofactor . We confirmed that the *torCAD* operon is directly regulated by the heme-containing protein TorC and is indirectly regulated by ArcA and by the availability of iron via active FNR and Fur, both regulatory proteins that influence the synthesis of the molybdenum cofactor. Furthermore, we identified a novel regulation mode of *torCAD* expression that is dependent on cellular levels of bis-MGD and is not used by other bis-MGD-containing enzymes like nitrate reductase.

**IMPORTANCE:**

In bacteria, molybdoenzymes are crucial for anaerobic respiration using alternative electron acceptors. FNR is a very important transcription factor that represents the master switch for the expression of target genes in response to anaerobiosis. Only *Escherichia coli* trimethylamine-*N*-oxide (TMAO) reductase escapes this regulation by FNR. We identified that the expression of TMAO reductase is regulated by the amount of bis-molybdopterin guanine dinucleotide (bis-MGD) cofactor synthesized by the cell itself, representing a novel regulation pathway for the expression of an operon coding for a molybdoenzyme. Furthermore, TMAO reductase gene expression is indirectly regulated by the presence of iron, which is required for the production of the bis-MGD cofactor in the cell.

## INTRODUCTION

Respiration is central to the metabolism of many microorganisms. However, there are many ecological niches in which the oxygen tension is low or oxygen is entirely absent. In the absence of oxygen, microbial life flourishes due to the ability of some microbes to grow by fermentation or by respiration via alternative electron acceptors. For example, trimethylamine-*N*-oxide (TMAO) is an alternative electron acceptor that is used by *Escherichia coli* (1).

The *E. coli* TMAO respiratory proteins encoded by the *torCAD* operon (2) face the periplasm and encompass a menaquinol or demethylmenaquinol electron donor (3, 4), the membrane-associated *c*-type cytochrome protein TorC and the molybdenum cofactor (Moco)-containing terminal reductase TorA responsible for substrate binding and conversion (2). TorA coordinates the Moco in the form of a bis-molybdopterin guanine dinucleotide (bis-MGD) cofactor and, therefore, belongs to the DMSO reductase family of molybdoenzymes (5). Molybdoenzymes of this family are only present in prokaryotes (6).

The synthesis of Moco and bis-MGD is a well-understood process, which is divided into four main steps in prokaryotes (7, 8), namely, (i) the conversion of 5′-GTP to cyclic pyranopterin monophosphate (cPMP) that is catalyzed by the MoaA and MoaC proteins (9–11); (ii) the insertion of two sulfur atoms into cPMP and formation of molybdopterin (MPT) that is catalyzed by MPT synthase composed of the (MoaD/MoaE)_2_ heterotetramer (12, 13); (iii) the insertion of molybdate into MPT and formation of Mo-MPT that is catalyzed by the MogA and MoeA proteins (14, 15). Produced Mo-MPT can be further modified, either by the action of the MocA protein that adds CMP to Mo-MPT, resulting in the formation of the MPT cytosine dinucleotide (MCD) cofactor present in enzymes of the xanthine dehydrogenase family (16), or by MobA that adds GMP to bis-Mo-MPT, resulting in the formation of the bis-MGD cofactor (17–19). Among these proteins, MoaA is a 2× [4Fe-4S] cluster-containing protein that directly links Moco biosynthesis to the assembly of iron-sulfur (Fe-S) clusters.

**Fig 1:**
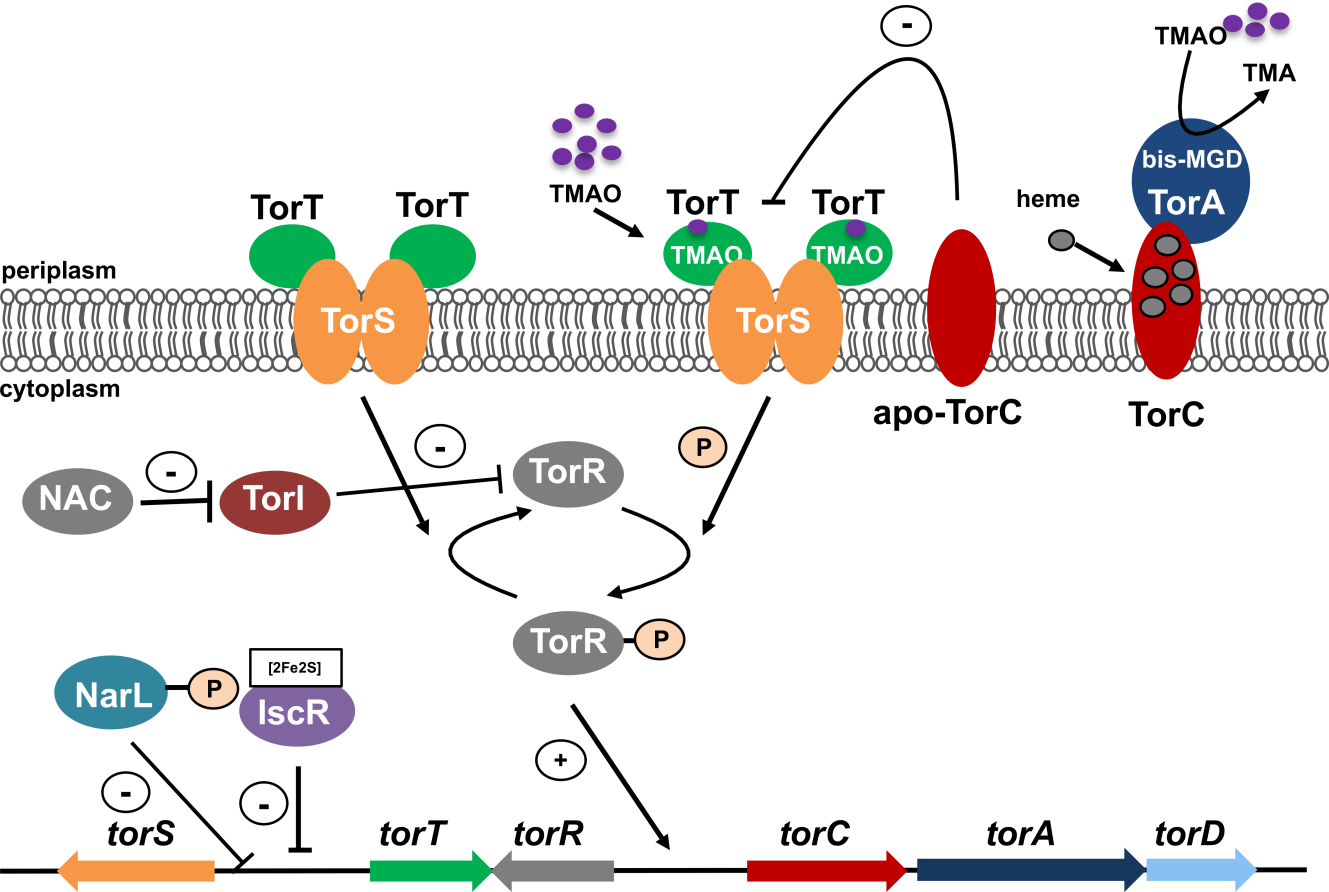
Model for the regulation of TMAO reductase in *E. coli* under anaerobic conditions. Details of the regulation of the *torCAD* operon and TorT and TorS are given in the text.

Many alternative respiratory molybdoenzyme systems employ the bis-MGD cofactor as the active site of substrate reduction, including nitrate reductases, DMSO reductases, and formate dehydrogenases (20). Generally, these operons are typically expressed under anaerobic conditions and are regulated by the transcriptional factor for fumarate and nitrate reductase (FNR) (21). FNR is a Fe-S cluster binding transcriptional regulator sensing O_2_ levels (22–24). Interestingly, TMAO reductase was described to be among the few molybdoenzymes for which the operon expression was shown to be independent from FNR (21, 23, 25, 26), but also by which its regulation of expression in *E. coli* was shown to be complex (Fig. 1). TMAO induces the transcription of the *torCAD* operon via a three-protein system: first, TorT senses the presence of TMAO in the periplasm (27), next, TorS interacts with TorT, transmits this signal across the membrane, and phosphorylates TorR (28), and finally, phosphorylated TorR, in turn, binds to the *torCAD* promoter region and activates its expression (29). Concomitantly, TorC heme cofactor binding is required, since otherwise, its apo form interacts with TorS to prevent TorR phosphorylation, thereby negatively regulating *torCAD* expression (28). Lastly, the expression of *torCAD* was shown to be regulated by TorI, nitrogen assimilation control (NAC) regulator, and NarL (30–32).

In the presence of oxygen, *E. coli* TMAO reductase operon expression is regulated by the transcriptional regulator IscR, which binds to a shared regulator site between *torT* and *torS* and represses their transcription (33–35) (Fig. 1). Cellular IscR levels are oxygen-sensitive, with the concentration of IscR being higher under aerobic conditions, which consequently reduces *torCAD* expression (33, 36). Moreover, it was shown that under aerobiosis, *torCAD* transcription was not uniform within a population, while the mean activity was similar to that observed under anaerobiosis. This mechanism has been referred to as “bet hedging” and has been suggested to facilitate a rapid adaptation of bacteria toward anaerobiosis in the presence of TMAO (33, 37).

IscR is a [2Fe-2S] cluster containing transcription regulator that was shown to predominantly regulate the expression of the *isc* and *suf* operons in *E. coli* (36, 38, 39). In its [2Fe-2S] cluster-bound form, IscR represses its own expression in addition to that of the *iscSUA-hscBA-fdx-iscX* operon and numerous additional genes in *E. coli* (40). In contrast, in its apo-form, IscR activates the expression of the SUF system (41). This mechanism allows IscR to fine-tune the Fe-S cluster synthesis in response to the presence of synthesized Fe-S clusters in the cell. Since the first and second steps of Moco biosynthesis depend on the presence of [4Fe-4S] clusters and on IscS, respectively, a downregulation of the *iscRSUA-hscBA-fdx-iscX* operon by IscR consequently will affect the cPMP synthesis and the sulfur transfer for the conversion of cPMP to MPT (21). For Moco biosynthesis, there are several other regulatory transcription factors that regulate the expression of operons coding for molybdoenzymes and Moco biosynthesis proteins (21). Among them are NarL (31, 42–44), NarP (45, 46), ArcA (47), NAC, and indirectly Fur and FNR (48, 49). The ferric uptake repressor (Fur) regulates the expression of iron homeostasis genes in response to intracellular iron levels. Since the first step of Moco biosynthesis depends on the presence of [4Fe-4S] clusters in MoaA (50), Moco biosynthesis subsequently depends on the presence of iron and Fe-S clusters and consequently on the regulators Fur, FNR, and IscR, as reported previously (21, 32).

FNR activates the transcription of the *moaABCDE* operon (32, 51), while FNR has been shown to act as a transcription repressor of the *moeAB* operon that is activated by phosphorylated ArcA (52). The expression of FNR itself is regulated by Fur. Fur is involved in the control of the intracellular iron level (53, 54). Additionally, Fur has been shown to be involved in the regulation of Fe-S cluster biosynthesis by negative regulation of the *sufABCDE* operon (55) and repression of the small RNA *ryhB* (56). At the transcriptional level, the regulators ModE, FNR, and ArcA ensure that transcription of the genes coding for Moco biosynthesis proteins only occurs under specific conditions (e.g., anaerobic growth conditions as sensed by FNR and ArcA) and is capable of producing active Moco (e.g., sufficient molybdate levels as sensed by ModE) (51). Additionally, the transcription of the genes of the *moa*-operon emphasizes an apparent feedback regulation by an accumulation of MPT in the cell via the Moco riboswitch (57, 58).

In this report, we reinvestigated the regulation of *torCAD* expression under anaerobiosis. We identified the bis-MGD cofactor as a requisite component to produce active TorA. While so far, it was believed that the expression of *torCAD* escapes FNR regulation (25), we could confirm that *torCAD* is not directly regulated via FNR, rather an indirect regulation circuit was identified as dependent on the availability of iron, involving active ArcA, IscR, FNR, and Fur, each that regulates the expression of operons coding for molybdenum cofactor synthesis proteins (21). Thereby, ArcA is indirectly involved in the regulation of *torA* expression by regulating the Moco content in the cell under aerobic conditions. Unexpectedly, we were able to identify a direct correlation between the presence of the bis-MGD cofactor and the expression of the *torCAD* operon. In Moco-deficient mutants, the TorA protein abundance was revealed to be largely reduced. The absence of bis-MGD affects *torCAD* expression by a so-far unknown mechanism.

## RESULTS

### Proteomic analysis of protein abundance under iron-limiting conditions

**Fig 2:**
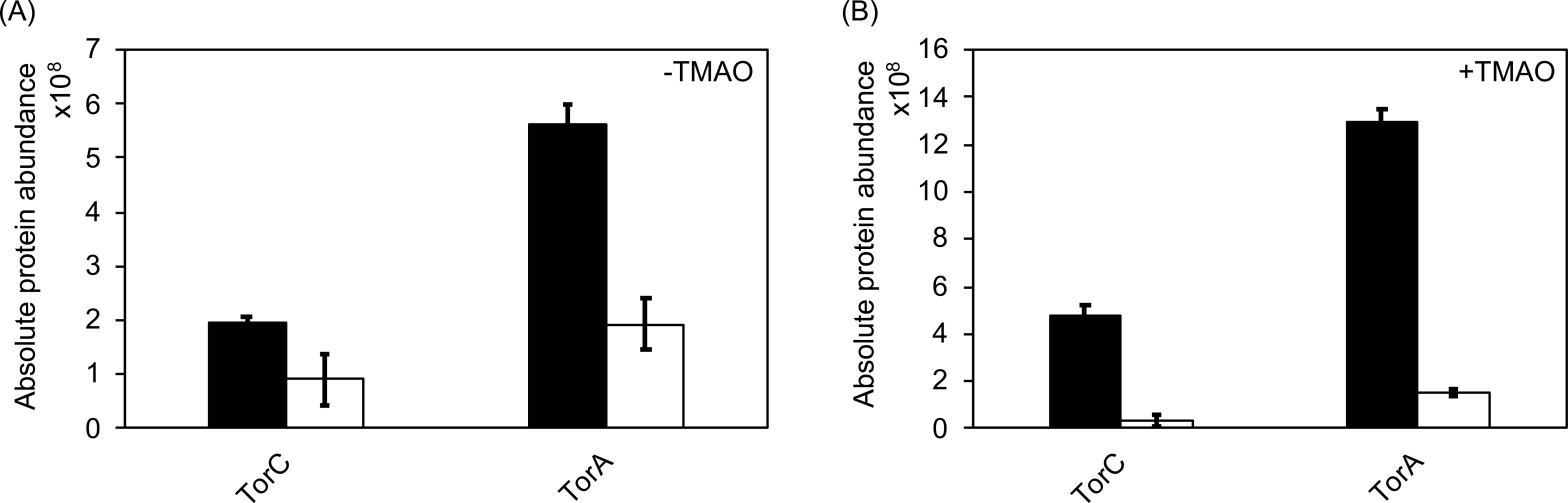
Iron-dependent production of TorA and TorC. (A and B) Absolute quantification of TorC and TorA proteins by proteomics (**A**) in the absence and (**B**) in the presence of 20 mM TMAO during growth. Cells were grown anaerobically in Luria Broth (LB) medium in the absence (black bars) or presence of 150 µM 2,2-DIP (white bars). A total of 25 µg of the purified peptides extracted from BW25113 cells was labeled with TMT10plex Isobaric Label Reagent followed by quantification by mass spectrometry. Three biological replicates were used to calculate standard deviations.

Previously, the presence of 150 µM dipyridyl (2,2-DIP) in the medium was shown to downregulate the expression of almost all operons coding for molybdoenzymes, based on an inactive FNR under these conditions (32). One operon coding for a molybdoenzyme, which had escaped previous attention, was the *torCAD* operon since it had been described to not be regulated by FNR. However, after a more critical reanalysis of the proteomic data, we were surprised to identify that TorCA abundance is largely reduced in the absence of iron and TMAO (Fig. 2A) and that this downregulation apparently is stronger than the reported induction in the presence of TMAO in the medium, since in the presence of TMAO and the absence of iron, the abundance of TorA and TorC was even more reduced (Fig. 2B). The altered abundance of other proteins largely influenced by the absence of iron included those the expression of which are activated by FNR, repressed by Fur, or regulated by RyhB, such as FepA, FhuA, EntF, FeoB, and YncE. These observations showed the reliability of our proteomics data under iron-limiting conditions and also illustrated that no other metals are chelated by DIP (Tables S2 to S5). For clarity, only new data and no repetitions from previously published work are depicted in Tables S2 to S5 (32). The abundance of ArcA, another global regulator, was also described to be lowered under iron-limiting conditions, based on the higher abundance of FNR and Fur [Table S1; Fig. 5 in reference (32)]. The lower abundance of TorCA under iron-limiting conditions is based on a lower transcription of these genes, as revealed by a transcriptional *torC-lacZ* fusion, which showed their completely downregulated expression in the presence of 150 µM 2,2-DIP (Fig. S1).

### ArcA and FNR do not bind to the *torCAD* promoter region

**Fig 3:**
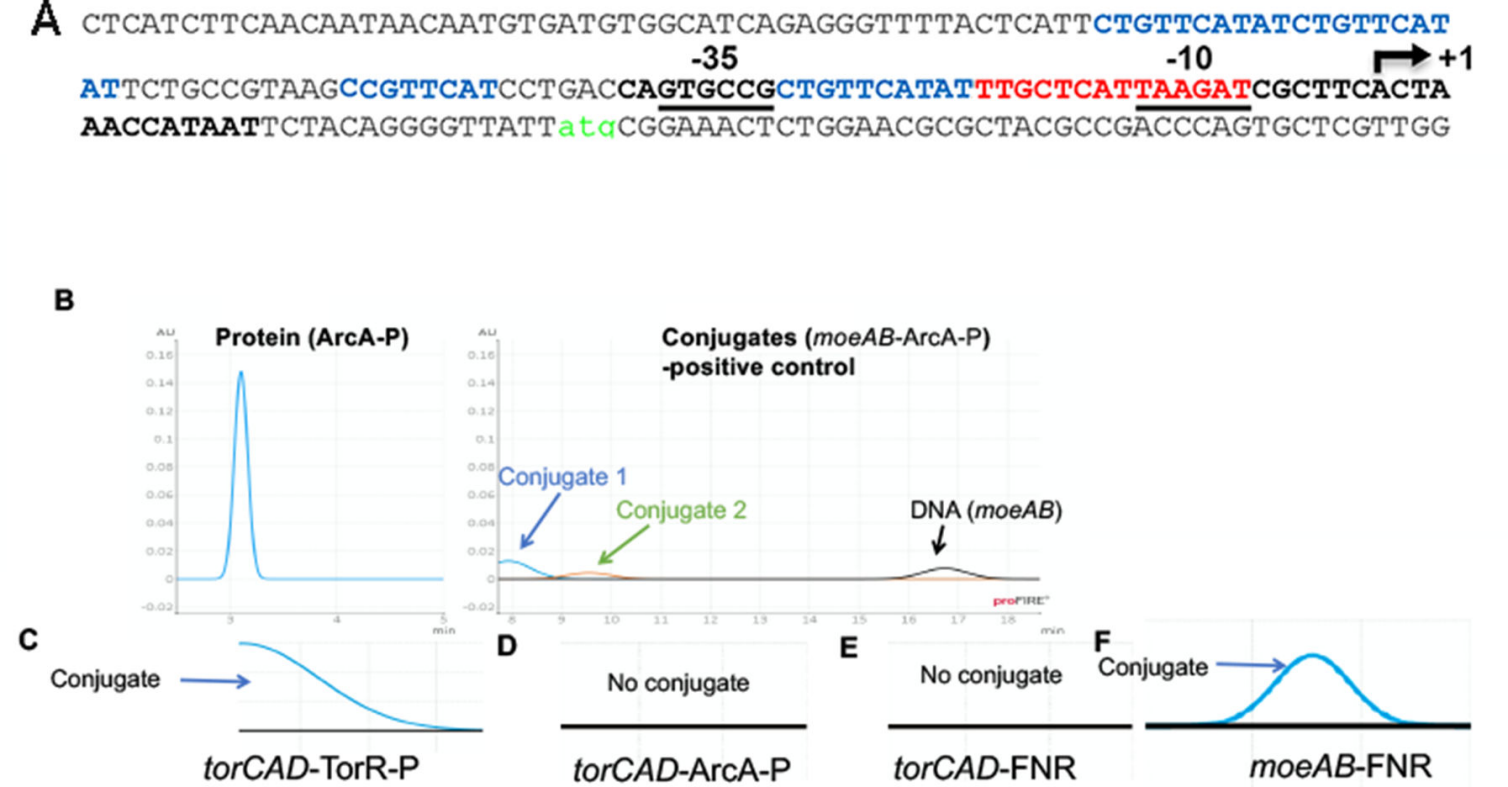
Potential binding sites of FNR, ArcA-P, and TorR-P in the *torCAD* promoter region. (**A**) Predicted/proven binding sites of FNR (red) and ArcA/TorR (blue). (**B**) A complete chromatogram from proFIRE for the conjugation of ArcA-P binding to *moeAB* promoter was used as a positive control. The protein ArcA-P peak represents the protein purity measured by the proFIRE. The two conjugate peaks represent the two possible conjugates formed, which could be due to ArcA-P binding to the promoter sequence in two different stoichiometries, or perhaps simply due to ArcA-P having two binding sites. The last peak is the free DNA peak in the sample injected. (**C**), (**D**), (**E**), and (**F**) represent the conjugate region of the chromatograms, for *torCAD*-TorR-P, *torCAD*-ArcA-P, *torCAD*-FNR, and *moeAB*-FNR binding, respectively.

Since the reduced abundance of TorC and TorA in the absence of iron parallels all other molybdoenzyme operon expressions that are regulated by FNR (21, 32), and since FNR and ArcA abundance was altered under these conditions, we wanted to confirm the presence or absence of binding sites within the *torCAD* promoter region for TorR, ArcA, and FNR. According to the consensus sequence defined for ArcA binding, there are putative motifs within the *torC* promoter (Fig. 3A, blue text) (47). However, since ArcA and TorR belong to the same family of transcriptional activators, the identified binding sites for ArcA might overlap with those previously identified for TorR (47, 59). Additionally, we identified a low-homology binding motif for FNR (Fig. 3A, red text) (47). The described FNR binding consensus sequence [TTGAT (nnnn)ATCAA] (60), however, overlaps quite well with the −10 RNA polymerase binding site within the *torC* promoter region, identified as TTGCT (nnnn)AAGAT. To investigate the binding of FNR, TorR, and ArcA to the predicted binding sites, we employed proFIRE for a qualitative understanding of the protein binding. This is a sensitive, surface plasmon resonance (SPR)-based method for protein-DNA interactions. ArcA was phosphorylated prior to binding experiments to obtain its active form (see Materials and Methods). Since ArcA-P was previously shown to bind to the *moeAB* promoter, we used this promoter region to establish a positive control for ArcA-P binding (Fig. 3B). In Fig. 3B, we observed conjugation peaks, clearly indicating that ArcA-P was actively bound to the *moe*AB promoter. Laterally, TorR-P was observed to bind to the *torCAD* promoter region to the predicted binding sites, since a conjugation peak appeared in the chromatogram (Fig. 3C). When ArcA-P binding was tested within the *torCAD* promoter, no conjugation peaks were detected (Fig. 3D). FNR was also tested for binding with the *torCAD* promoter. We observed no conjugation peak (Fig. 3E), hence, no binding was observed. However, as a positive control, FNR binding to the *moeAB* promoter region was observed, showing the functionality of our purified FNR (Fig. 3F). Overall, TorR-P binding to the *torCAD* promoter could be confirmed and ArcA-P and FNR binding to this region could be excluded, while ArcA-P and FNR were binding to the *moeAB* promoter region, as reported previously (52).

### Analysis of the heme content as a source for the reduced TorCA abundance

Another factor that is influenced by the absence of iron is heme. Heme is essential for the activity of TorC. In the absence of heme, apo-TorC negatively influences the phosphorylation of TorR and subsequently impedes the transcription of the *torCAD* operon (Fig. 1) (28). While the expression of the genes coding for enzymes of heme synthesis is regulated by FNR (61), the level of heme might also be reduced due to the iron limitation caused by the addition of 2,2-DIP. The expression of the *ccm* operon is activated by FNR and, therefore, regulated by Fur and ArcA, which regulate FNR expression (Fig. S2).

Therefore, we analyzed the porphyrin content in strains Δ*nac,* Δ*arcA,* Δ*ccmA,* Δ*fnr,* Δ*fur, and* Δ*torI* (Fig. S3). Overall, no major changes in the heme porphyrin content were observed for either the Δ*nac* or Δ*arcA* strains, and only a slight reduction in the heme porphyrin content was observed for the Δ*torI* strain. As shown in Fig. S3, the heme porphyrin content was mainly reduced in the Δ*ccmA* and Δ*fur* strains, showing that the reduction of TorCA abundance is not only based on a reduced heme content due to the absence of active FNR. Thus*,* other extant factors appear to downregulate *torCAD* expression under iron-limiting conditions.

### Analysis of the iron-dependent expression of *iscR* under anaerobiosis in the presence of TMAO

**Fig 4:**
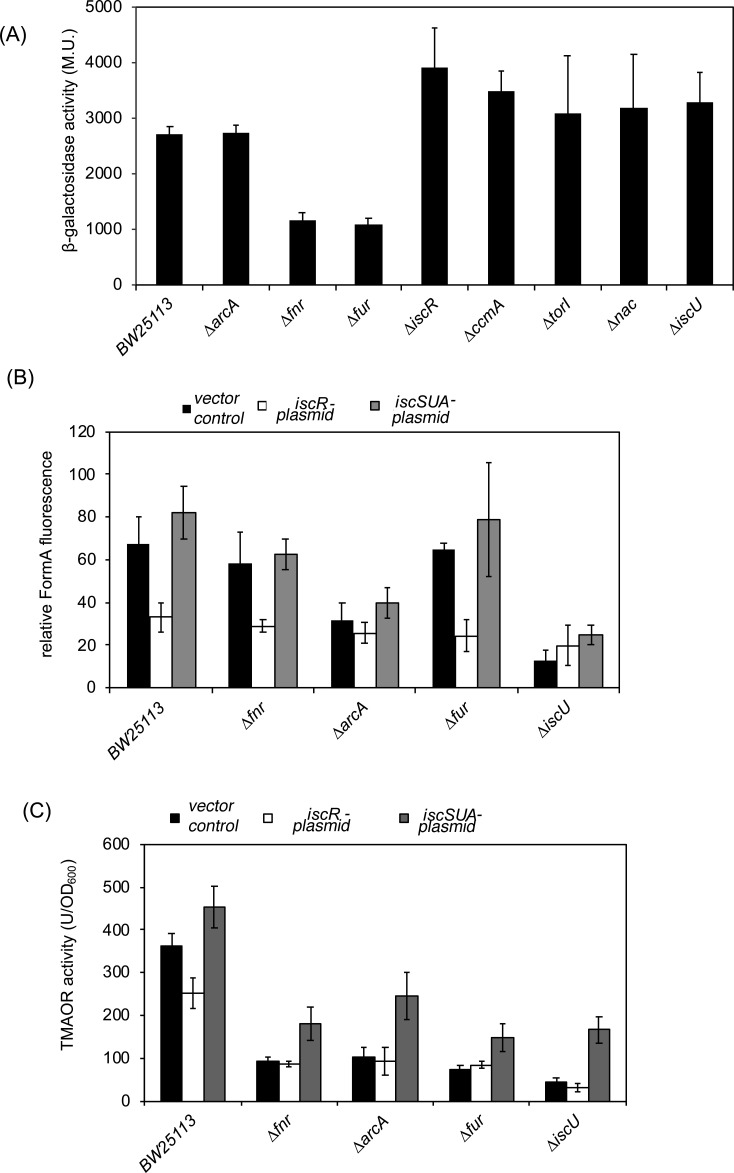
Expression of a *PiscR-lacZ* fusion TorA activity and Moco content in *E. coli* mutant strains. (**A**) β-galactosidase activities expressed in Miller units were determined for *iscR-lacZ* fusion in *E. coli* BW25113 wild-type strain and Δ*nac*, Δ*fur*, Δ*torI*, Δ*arcA*, Δ*ccmA*, Δ*IscU*, Δ*fnr*, and Δ*iscR* strains. The strains were cultivated anaerobically in the presence of 20 mM TMAO for 4 h. β-galactosidase activities were normalized to the OD_600 nm_ values. Standard deviation was calculated from three biological replicates. (**B**) Quantification of relative amounts of Moco in the *E. coli* BW25113 wild-type strain and the mutant Δ*fnr*, Δ*arcA*, Δ*fur*, and Δ*iscU* strains. The strains were grown anaerobically in the presence of 20 mM TMAO for 8 h in the LB medium. Total Moco in crude extracts was oxidized overnight with acidic iodine into its fluorescence derivative, FormA. FormA was separated and quantified by its fluorescence monitored at *λ*_ex_ = 383 nm and *λ*_em_*=* 450 nm. The black, white, and gray bars represent without the *isc* operon, with *iscRSUAhscABfdx iscX*, and with *iscSUAhscABfdx-iscX*, respectively. (**C**) TMAO reductase activity in the *E. coli* BW25113 wild-type strain and the mutant Δ*fnr*, Δ*arcA*, Δ*fur*, and Δ*iscU* strains. The strains were cultivated anaerobically in the presence of 20 mM TMAO. TMAO reductase activities (in units) were normalized to the OD_600 nm_ values. Standard deviation was calculated from three biological replicates. The black, white, and gray bars represent with either the vector only, or with a plasmid carrying the *iscRSUAhscABfdx-iscX* or *iscSUAhscABfdx-iscX* genes, respectively.

The production of *E. coli* TMAO reductase is negatively regulated by the transcriptional regulator IscR in the presence of oxygen (33–35) (Fig. 1). Under iron-limiting conditions, IscR upregulates its own expression (as obvious from the proteomic data Tables S2 to S5), which consequently results in a lower *torCA* expression. Clearly, IscR could be one factor for the lower abundance of TorC and TorA, but a lowered cellular Fe-S cluster abundance results in increased amounts of IscR. To test this, we first analyzed the expression of an *iscR-lacZ* fusion in different mutant strains to assess the abundance of the IscR protein in these strains. We analyzed the expression of an *iscR-lacZ* fusion in the BW25113 parental strain and in Δ*arcA,* Δ*fnr,* Δ*fur,* Δ*iscR,* Δ*ccmA,* Δ*torI,* Δ*nac,* and Δ*iscU* mutant strains under anaerobic conditions in the presence of TMAO (growth curves for some of these strains are shown in Fig. S4). The obtained β-galactosidase activities showed that, as expected, a slightly increased *iscR-lacZ* expression was obtained in the Δ*iscR,* Δ*ccmA,* Δ*torI,* Δ*nac,* and Δ*iscU* strains, confirming that apo-IscR activates its own expression also under anaerobic conditions (Fig. 4A). In contrast, in the Δ*fnr* and Δ*fur* mutants, the expression of *iscR* was 50% reduced and in the Δ*arcA* mutant, it was unaffected (Fig. 4A). This confirms that also under our conditions, the expression *iscR* is influenced by the availability of Fe-S clusters, which is consistent with previously published data (62). To analyze whether the Fe-S cluster levels and/or the presence of IscR affect the TorCA system, we expressed the *iscSUA-hscBA-fdx-iscX* operon (more Fe-S clusters) and the *iscR-iscSUA-hscBA-fdx-iscX* operon (more IscR and less Fe-S clusters) in strains Δ*fnr,* Δ*arcA,* Δ*fur,* Δ*iscU*, and analyzed the Moco content (Fig. 4B) and the TorA activity (Fig. 4C) in the respective strains. The results depicted in Fig. 4B and C show that TorA activity and the Moco content are increased after overexpression of the *iscSUA-hscBA-fdx-iscX* operon, whereas when *iscR* was additionally overexpressed, it resulted in a reduction of Moco content and TorA activity. This shows that the cellular Fe-S cluster concentration influences the Moco content, which might be one crucial factor for the reduced TorA activity. However, how the Moco content has an effect on TorA and TorC abundance needs further investigation.

### The influence of ArcA on TorA activity and Moco abundance

**Fig 5:**
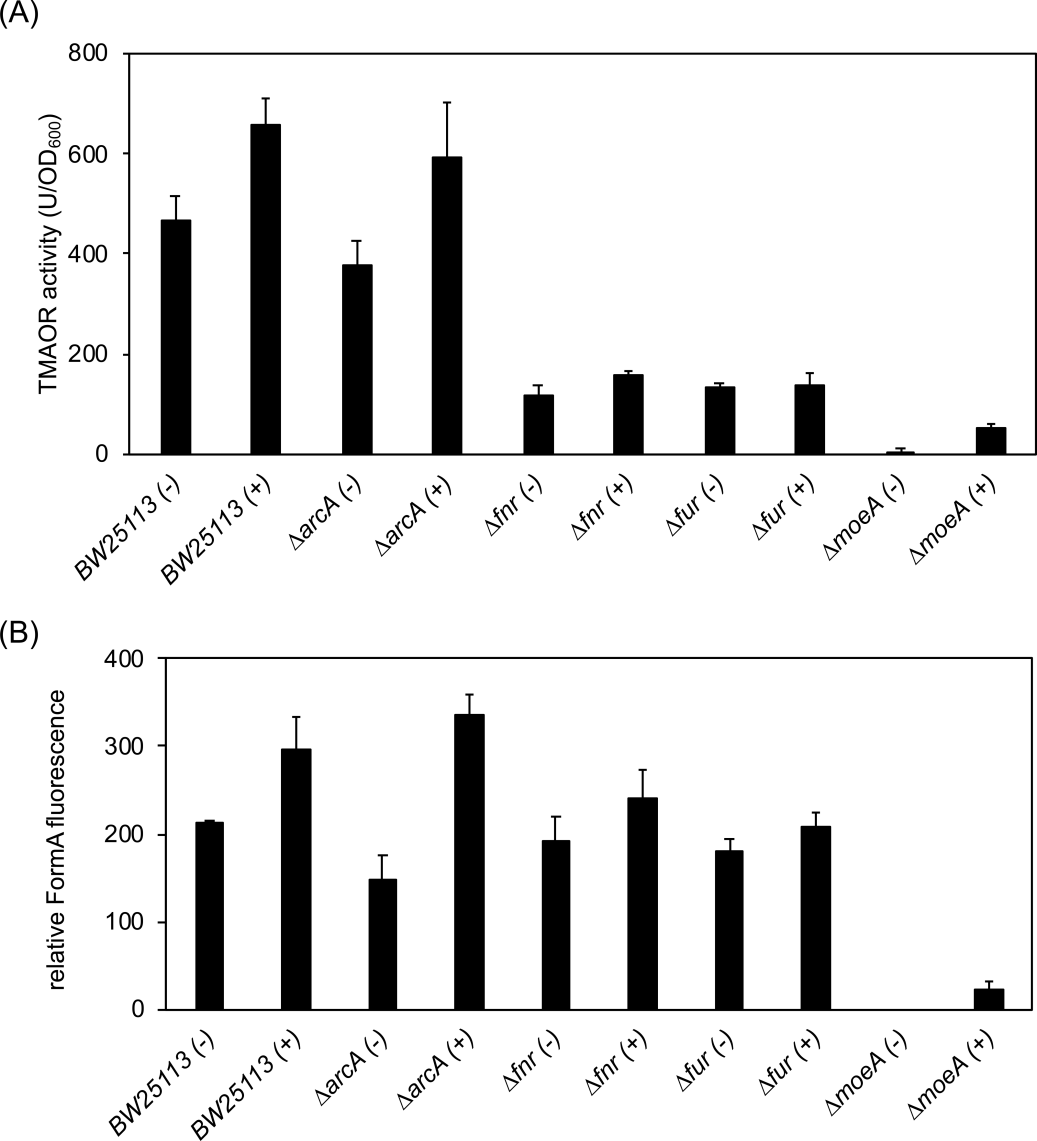
The influence of overexpression of *arcA* in different mutant strains in *E. coli*. (**A**) TMAO reductase activity in *E. coli* BW25113 wild-type strain and the mutant *ΔarcA*, *Δfnr*, and *ΔmoeA* strains. Activities (in units) were normalized to the OD_600 nm_ values. Standard deviation was calculated from three biological replicates. (+) and (−) denote the strains with and without an arcA overexpression plasmid. Protein production was induced by the addition of 1 mM IPTG. (**B**) Quantification of relative amounts of Moco in different mutant strains. The strains were grown anaerobically in the presence of 20 mM TMAO for 4 h in the LB medium. Total Moco in crude extracts was oxidized overnight with acidic iodine into its fluorescence derivative, FormA. FormA was separated and quantified by its fluorescence monitored at *λ*_ex_ = 383 nm and *λ*_em_ = 450 nm.

We reported previously that the abundance of ArcA is lowered in the presence of 150 µM 2,2-DIP (32). Since ArcA activates the *moe*A operon expression in addition to FNR, and since FNR regulates the *moaABCDE* operon expression, a lowered ArcA abundance might result in a lowered cellular Moco content. To investigate the role of ArcA on TorA activity and the cellular Moco content, we overexpressed *arcA* in BW25113 and in the Δ*fnr,* Δ*arcA,* Δ*fur,* and Δ*moeA* mutant strains. The results show that by the overexpression of *arcA*, the activity of TorA (Fig. 5A) and the Moco content (Fig. 5B) were increased in the BW25113 parental strain and in the Δ*moeA* mutant strain in comparison to the strains without additional ArcA present. This reflects a positive effect on the expression of the *moa*-operon that is positively regulated by FNR since higher FNR levels are obtained under these conditions. Therefore, we conclude that lower concentrations of ArcA result in a lower expression of genes involved in Moco biosynthesis, lowering the cellular Moco concentration. Surprisingly, no TorA activity was obtained in the Δ*moeA* mutant strain, but the activity could be slightly rescued by the expression of *arcA* in this strain, which resulted in an increase in Moco levels (Fig. 5B).

### Regulation of *torCAD* expression by the availability of Moco

**Fig 6:**
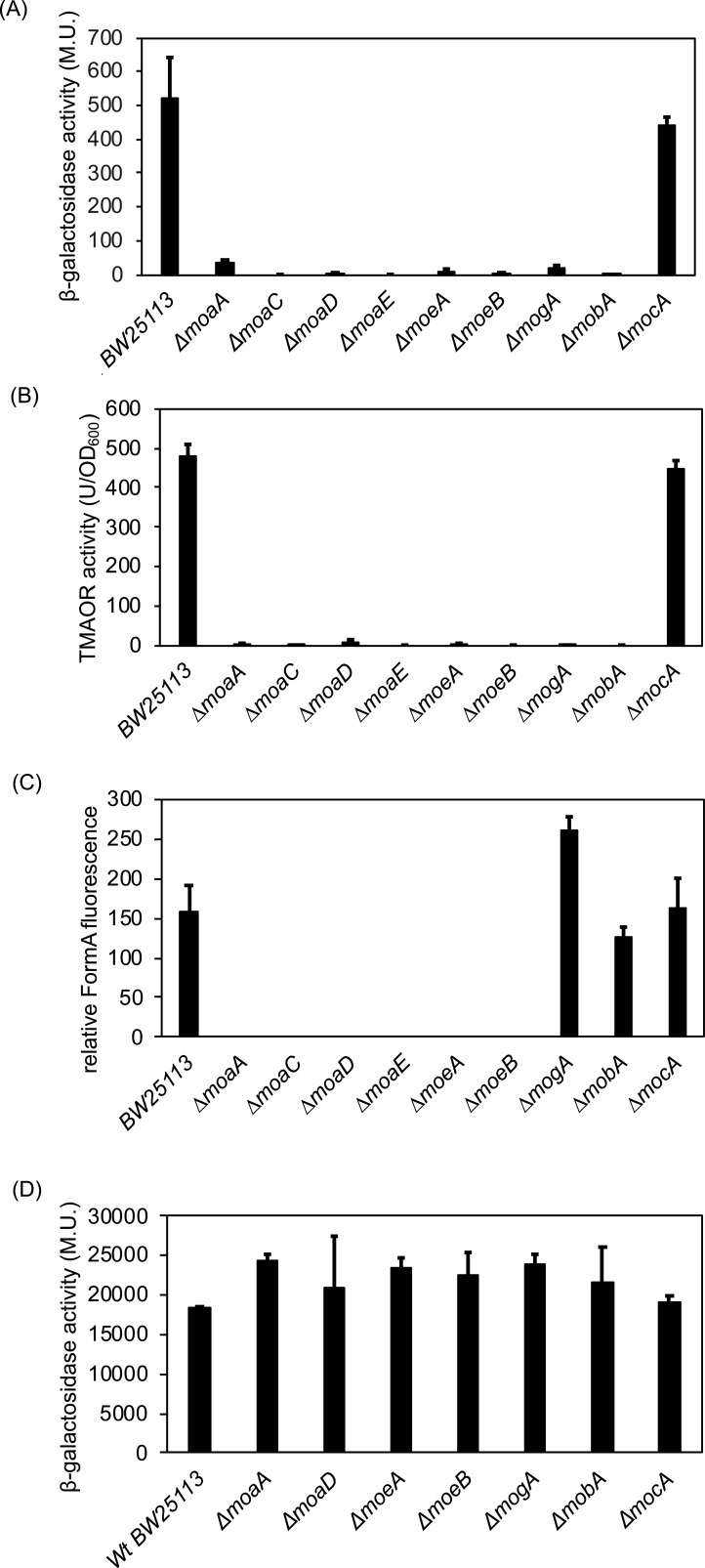
Moco-dependent expression of the *torC-lacZ* fusion, TorA activities, and Moco content. (**A**) β-galactosidase activities in Miller units were determined for the *torC-lacZ* fusion in Moco-deficient mutant stains. The indicated mutant strains were grown anaerobically in the presence of 20 mM TMAO. Standard deviations were calculated from three biological replicates. (**B**) TMAO reductase activity in different *E. coli* mutant strains. Bacteria were grown anaerobically in the presence of 20 mM TMAO. Standard deviations were calculated from three biological replicates. TMAO reductase activities were normalized to OD_600 nm_. (**C**) Quantification of relative amounts of Moco in different mutant strains. The strains were grown anaerobically in the presence of 20 mM TMAO for 4 h in the LB medium. Total Moco in crude extracts was oxidized overnight with acidic iodine into its fluorescence derivative, FormA. FormA was separated and quantified by its fluorescence monitored at *λ*_ex_*=* 383 nm and emission at *λ*_em_*=* 450 nm. (**D**) β-galactosidase activities in Miller units of the *narG-lacZ* fusion in Moco-deficient mutants. Bacteria were grown anaerobically in the presence of 20 mM KNO_3_. Standard deviations were calculated from three biological replicates.

The results above imply that the *torCA* abundance might be influenced by the availability of Moco, as revealed by a lack of TorA activity in the Δ*moeA* mutant strain. Therefore, we used additional mutant strains in Moco biosynthesis genes to analyze their effect on *torCAD* expression, TorA activity, and associated Moco content. The *torC-lacZ* fusion was introduced into the BW25113 parental strain and the Moco-deficient strains Δ*moaA,* Δ*moaC,* Δ*moaD,* Δ*moaE,* Δ*moeA,* Δ*moeB,* Δ*mogA,* Δ*mobA*, and Δ*mocA* and cells were grown under anaerobic conditions with 20 mM TMAO for 4 h (growth curves are shown in Fig. S4). In all Moco-deficient strains with the exception of the Δ*mocA* strain, only a residual expression of the *torC-lacZ* fusion was observed (Fig. 6A). While the *moaABCDE*, *moeAB*, *mogA*, and *mobA* gene products are involved in bis-MGD biosynthesis, MocA catalyzes the last step of MCD biosynthesis, the addition of CMP to Mo-MPT. This form of the cofactor is present in the *E. coli* periplasmic aldehyde oxidoreductase PaoABC enzyme but not in enzymes of the DMSO reductase family like nitrate reductase or TMAO reductase.

Additionally, we investigated the activity of TorA (Fig. 6B) in the same Moco-deficient mutant strains and compared them to the Moco content (measured as relative Form A fluorescence) (Fig. 6C). The results show a largely reduced activity of TorA in all tested Moco-deficient strains with the exception of Δ*mocA* (Fig. 6B), in which TorA activity was detectable to comparable levels as in the BW25113 parental strain. Overall, the obtained TMAO reductase activities correlated well with the obtained overall Moco content in the cell extracts of the corresponding mutant strains (Fig. 6C). As expected, in the Δ*mogA* and Δ*mobA* strains, Moco can be detected, since these strains either contain MPT or Mo-MPT, respectively (Fig. 6C). When using a different strain background (RK4353), we obtained the same lack of TorC expression in the RK4353Δ*moaA* mutant strain, showing that the effects are not based on the BW25223 strain background (Fig. S5).

To determine whether the lack of Moco affecting the expression of the *torCAD* operon is a common effect among other operons coding for bis-MGD containing molybdoenzymes in *E. coli*, we additionally analyzed the expression of a *narG-lacZ* fusion in the same mutant strains as a control (Fig. 6D). Transformed cell strains were grown under anaerobic conditions in the presence of 20 mM KNO_3_ for 4 h. As shown in Fig. 6D, no major changes in β-galactosidase activity were obtained in the Moco-deficient mutant strains in comparison to the BW25113 parental strain. Here, we propose that for *torCAD* expression*,* the bis-MGD cofactor seems to be mandatory, a regulation that is specific to this operon and not found for the expression of other operons coding for molybdoenzymes (57).

### *torC*-promoter deletions narrow down the potential bis-MGD binding site

**Fig 7:**
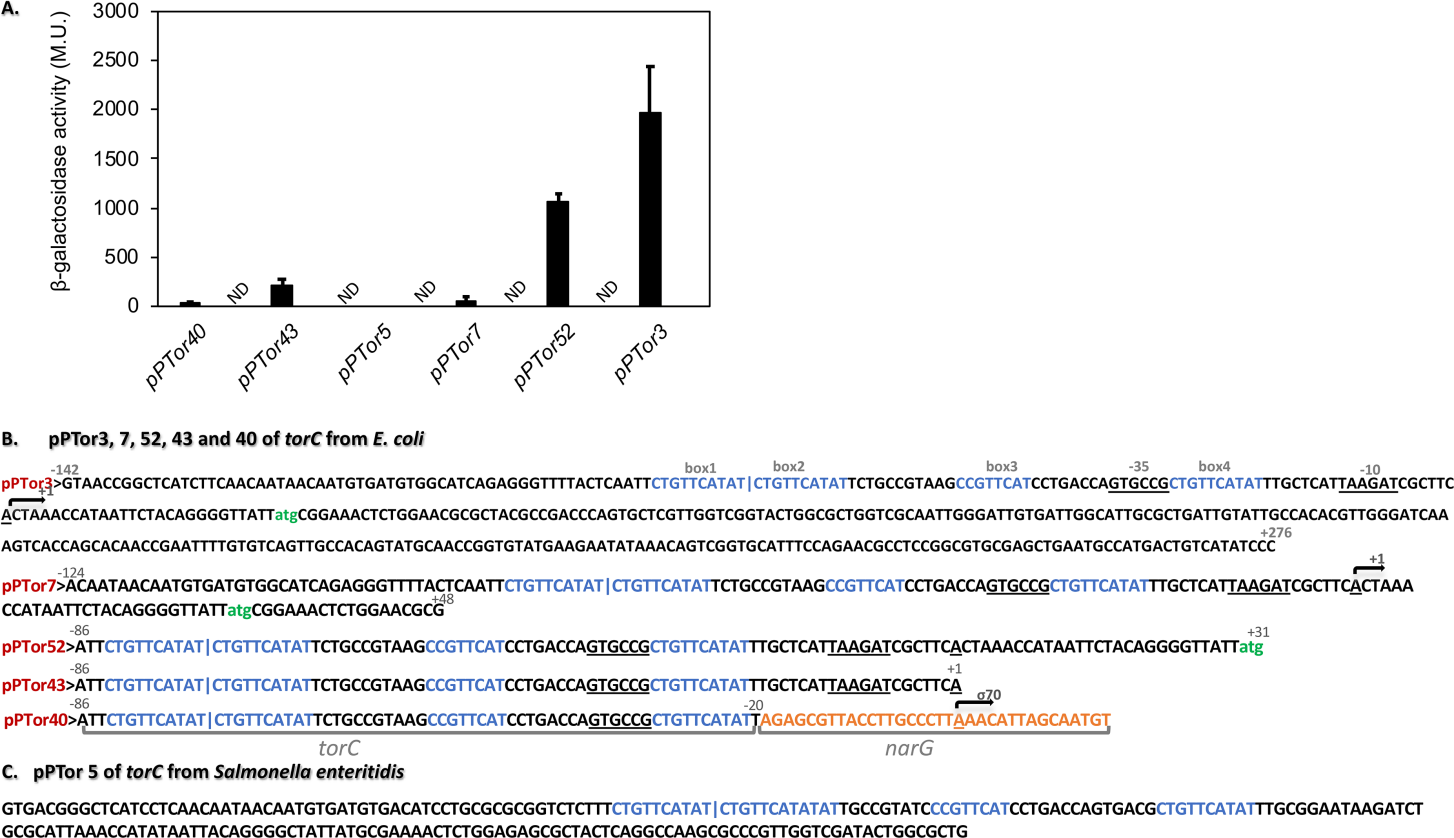
Effect of promoter deletions on the expression of *torCAD*. (**A**) β-galactosidase activity in the Miller unit was determined for *torC-lacZ* fusion in *E. coli* BW25113 (black bars) and the *ΔmoaA* mutant strain (white bars). The strains were cultivated anaerobically in the presence of 20 mM TMAO for 4 h. β-galactosidase activities were normalized to the OD_600 nm_ values. Standard deviation was calculated from three biological replicates. ND, no activity detectable. (**B**) Shown is the promoter region of pPTor3 and below the sequences of pPTor7, pPTor52, pPTor43, pPTor40, and pPTor5, which contains the region of *S. enteritidis*. pPTor40 is a fusion of the *ptorC* and *narG* promoter. The other fusions have the promoter region fused to *lacZ* in different lengths to narrow down the region of the potential riboswitch (all derivates of pPTor3).

Since the bis-MGD-dependent expression seems to be specific for the *torCAD* operon, we wanted to investigate this further, also to narrow down a potential bis-MGD-binding site in the promoter and after the +1 region of *torC*. In the case of *torCAD*, the bis-MGD cofactor is required for the induction of *torCAD* expression, likely at the level of transcription, since our *torC-lacZ* fusion is a transcriptional *lacZ* fusion. To narrow down a potential bis-MGD binding site, we analyzed different transcriptional fusions using 5′ and/or 3′ truncated *torC* regions fused to *lacZ*. We could identify five *torC-lacZ* fusions in which the induction of gene expression was either lost or largely reduced in comparison to the unmodified sequence in the pTor3 fusion (Fig. 7A). The transcriptional start site had been mapped previously and is marked as +1 in the promoter sequence (Fig. 7B) (2). For clarity, pTor40 is a fusion of the −35 sequence of the *torC* promoter and the −10 region of the *narG* promoter. However, with pTor40, almost no β-galactosidase activity was obtained. Similarly, when the promoter region of *Salmonella enteritidis* was used (pPTor5), no β-galactosidase activity was obtained, showing that the observed regulation is specific to *E. coli torCAD*. Furthermore, the binding site seems to be located between +1 and +31 and extending after +48, since in pTor43 and pTor7, the induction of the *torC* transcription was largely reduced, while in pTor52, only a 50% reduced activity was observed, making it likely that the binding site is extended after nucleotide +48. In the Δ*moaA* strain, no activity was detectable for all promoter deletions (ND in Fig. 7A). We conclude that the bis-MGD binding site might be located in the 5′ untranslated region (UTR) after the transcriptional start site, making a bis-MGD DNA-binding protein unlikely to bind as a regulatory factor.

## DISCUSSION

Herein, we identified a novel regulation of the *torCAD* operon by the presence of the bis-MGD cofactor that is essential for the transcription of the *torCAD* operon. Since the biosynthesis of Moco requires the presence of Fe-S clusters to generate functionally competent biosynthetic proteins, the expression of the *torCAD* operon is also dependent on the availability of iron in the cell. So far, the expression of the *torCAD* operon has been described to be positively regulated by TorR (63) and negatively regulated by NarL (31). Furthermore, apo-TorC lacking heme was shown to interact with TorS to result in dephosphorylation of TorR and, thus, to result in a lowered transcription of the *torCAD* operon (28) (Fig. 1). However, herein we could exclude that the lowered *torCAD* expression under iron-limiting conditions is based on the absence of heme. In contrast to other alternative respiratory systems, the expression of *torCAD* is not completely repressed by the presence of oxygen (2). In aerobically grown cells, the *torCAD* operon is expressed; however, the activity of TMAO reductase was reported to be largely reduced down to 5% as compared to anaerobically grown cells (64). In other recent reports, it was shown that the expression of the genes encoding the regulators TorT and TorS is regulated by IscR (27, 33, 65). IscR binds to the promoter region between *torT* and *torS* and represses the transcription of both genes (33). IscR is more abundant during aerobic growth than during anaerobic growth, which suggests that the increased expression of TorT and TorS under anaerobic conditions is a result of the decreased IscR concentration. The negative IscR regulation of the *torCAD* operon was confirmed in our study to act indirectly on *torCAD* expression by a reduction of Fe-S cluster biosynthesis and consequently by a reduction of Moco production in the cell.

While TorA functions as an alternative electron acceptor under anaerobic conditions, it has been assumed so far that *torCAD* expression escapes the regulation by FNR (23, 66, 67). This has always been considered as surprising, since FNR regulates the expression of most of the genes coding for molybdoenzymes in *E. coli*, like the *narGHJI* operon (68, 69), the *dmsABC* operon (69), the *napFDAGHBC* operon (44), the *xdhABC* operon (70), the *ynfEFGH* operon (71, 72), the *ydhYVWXUT* operon (72) and the *fdnGHI* operon (73).

In this report, we confirm that the regulation of the *torCAD* operon by FNR does not occur directly at the transcriptional level, but we reveal an indirect reduction in the absence of active FNR, by a reduction of the cellular Moco levels. In a report by Myers and coworkers, the regulation of genes by FNR has been categorized into seven categories, which include direct and indirect regulation by FNR (60). We think that the regulation of the *torCAD* operon falls into category 6, reflecting indirect FNR regulation through hierarchical transcription regulator action that includes ArcA.

In this study, we investigated the regulation of the *torCAD* operon by the availability of iron and the molybdenum cofactor. We were able to show that the *torCAD* operon is indirectly regulated by FNR through ArcA and that both regulate the expression of the *moeAB* and the *moaABCDE* operons. In addition, we observed an indirect iron availability regulation mode via the availability of Fe-S clusters through IscR, Fur, and heme. IscR serves as a repressor of *torT*, *torS*, and *iscSUA-hscAB-fdx-iscX* operon expression. When the *iscS* operon was overexpressed in the absence of IscR, an increased TorA activity was obtained. The presence of IscR inhibited the expression of *torCAD* through its role as a repressor. Additionally, a lowered Moco content is present, and we were able to reveal a novel regulation of *torCAD* expression by the cellular availability of Moco, in particular, in the form of bis-MGD. Furthermore, FNR and ArcA are required for the expression of the *moeAB* operon. All these factors jointly contribute to a complete downregulation of *torCAD* expression in the absence of iron (mimicked by the addition of 2,2-DIP) by a reduction of the cellular Moco content.

Herein, the novel regulation mode observed for *torCAD* expression is dependent on Moco availability, contrasting with the regulation of other molybdoenzymes in *E. coli*. Regulation of gene expression via Moco availability apparently allows for a fine-tuning of the synthesis of TMAO reductase under anaerobic conditions, since only when Fe-S clusters, heme, and Moco are present, the *torCAD* operon is optimally expressed in the absence of oxygen. This complex regulation might be advantageous for the cell to save energy, and the synthesis of the molybdenum cofactor is further also dependent on the presence of [4Fe-4S] clusters bound to the MoaA protein that catalyzes the first step of Moco biosynthesis, the conversion of 5′-GTP to cPMP.

Under anaerobic conditions, we identified an indirect effect on *torCAD* expression by the transcriptional regulator ArcA. Since ArcA does not directly bind to the *torCAD* promoter, we conclude that the observed reduction in the expression in the Δ*arcA* mutant strains is based on an indirect regulation dependent on the synthesis of active Moco in the cell. It has been revealed previously that ArcA-P plays a role in the regulation of Moco biosynthesis, by activating the expression of the *moeAB* operon (also regulated via FNR) (51, 74). A lack of ArcA, therefore, will lead to lowered amounts of Moco in the cell, which then results in a downregulation of *torCAD* transcription. Indeed, in Δ*arcA* cells, the concentration of Moco was lowered as compared to wild-type cells (Fig. 5B).

Unexpectedly, we identified a regulation of *torCAD* expression that is dependent on the synthesis of the bis-MGD cofactor in the cell. In all mutant strains involved in the biosynthesis of it, no expression of the *torCAD* operon was detected and additionally, the TorA protein abundance was largely reduced in these mutant strains. In contrast, a mutant strain with a defect in MCD biosynthesis showed no effect on *torCAD* expression. Furthermore, in a *mogA* mutant strain, which still contains MPT, *torCAD* expression was observed, pointing to the bis-MGD cofactor as a regulatory metabolite and not any of the other Moco molecules.

One explanation for the reduced expression of the *torC-lacZ* might be the presence of a bis-MGD-dependent riboswitch upstream of the *torC* gene. The absence of the bis-MGD cofactor would cause a different conformation of the mRNA in the 5′-UTR of *torC* that, consequently, results in either a terminated transcription or a block in the Shine-Dalgarno sequence, thereby inhibiting translation. We consider the transcriptional regulation with the access of the terminator sequence more likely since we investigated a transcriptional *torC-lacZ* fusion. Furthermore, by analysis via promoter deletions, we were able to narrow down the riboswitch to the 5′-UTR starting from the transcriptional start site and being extended even after the 5′ UTR to at least the first 20 bp within the translated region of TorC. The same regulation was not observed with promoter regions from other organisms, like *S. eneritidis*, so the riboswitch seems to be specific for *E. coli torC*. The other alternative is that apo-TorA might influence the phosphorylation of TorR by interfering with the TorS-TorR regulatory system (either by binding to TorS or to TorR). However, this has not been identified so far and is considered rather unlikely as a regulatory factor. Also, TorR does not bind the bis-MGD cofactor (data not shown). This implies that the *torCAD* operon might be regulated by a Moco riboswitch, as it has been reported previously for the *moaABCDE* operon. However, the sequences in front of the *moaA* coding region and the *torC* coding region are not conserved, pointing to differences in the regulatory molecule. This riboswitch sequence seems to be specific to the *torCAD* operon since a similar regulation of the *narG* operon has not been obtained. Since a riboswitch sequence has not been reported so far in the *torC* 5′-untranslated region and as the binding of the bis-MGD cofactor to RNA has not yet been confirmed, we will investigate the *torCAD* regulation by Moco in future studies with a particular focus on the bis-MGD cofactor binding to a potential riboswitch sequence. Overall, the riboswitch of the *torCAD* operon is different from the reported one of the *moaA* operon. Here, the riboswitch sequence is highly conserved among bacteria and was shown to act on the level of translation and be highly specific for Moco, since the tungsten analog Wco did not give the same regulatory output. However, in the case of the Moco riboswitch, the identity of the exact Moco-associated metabolite regulating the expression has not been identified to date. Herein, we could at least narrow down the potential riboswitch binding site from the 5′-UTR of *torC* between +1 and extending to the translated region after +48. This localization also makes regulatory DNA-binding proteins that respond to the cellular bis-MGD content unlikely to bind. We will investigate the bis-MGD cofactor binding site in the future in more detail.

## MATERIALS AND METHODS

### Bacterial strains, plasmids, media, and growth conditions

BW25113 (referred to as parental strain) and the isogenic mutant strains Δ*torA,* Δ*fnr,* Δ*fur,* Δ*nac,* Δ*torI,* Δ*narL,* Δ*ccmA,* Δ*arcA,* Δ*iscR,* Δ*modE,* Δ*moaA,* Δ*moaC,* Δ*moaD,* Δ*moaE,* Δ*moeA,* Δ*moeB,* Δ*mogA,* Δ*mobA,* Δ*mocA,* and Δ*iscU* were obtained from the Keio collection from the National BioResource Project (National Institute of Genomics, Japan) (75, 76). All strains and plasmids used in this study are listed in Table S1. Bacteria were grown anaerobically in closed Schott flasks in LB medium at 37°C in the presence of 20 mM TMAO and 0.1% glycerol. When required, 150 µg/mL ampicillin, 50 µg/mL kanamycin, or 50 µg/mL chloramphenicol was added to the medium during growth. As indicated, the iron chelator 2,2-DIP at a concentration of 150 µM was added to the medium. Bacteria were grown under anaerobic conditions for 4 h to (OD_600_ 0.5–1.5) until the mid-log phase was reached (growth curves of selected strains and in the presence of 150 µM DIP are shown in Fig. S4), collected and frozen in liquid nitrogen, and stored in −80°C for further experiments.

### Proteomic studies—peptide labeling and absolute quantification

Bacteria were grown anaerobically in LB medium until the mid-log phase. After washing in 50 mM Tris-HCl, pH 8.0, cells were pelleted, resuspended in the previously mentioned buffer, and sonicated. A total of 100 µg of cell extract was mixed with 8 M Urea in 10 mM Tris-HCl, pH 8.0 and loaded on filter columns (Microcon-30 kDa Centrifugal Filter Unit with Ultracel-30 membrane). Columns were washed with 8 M urea in 10 mM Tris-HCl, pH 8.0, reduced using 10 mM DTT in 8 M Urea, and alkylated using 27 mM iodoacetamide in 10 mM Tris-HCl, pH 8.0. Afterward, columns were mixed at 600 rpm in a thermomixer for 1 min and incubated without mixing for further 5 min. A total of 8 M urea in 10 mM Tris-HCl, pH 8.0 was added to each column and centrifuged. After this step, 14-h digestion with trypsin was performed. Reactions were stopped by the addition of 10% trifluoro acetic acid (TFA). Peptides were purified on C18 SepPack columns (Teknokroma) and eluted with 800 µL of 60% acetonitrile (ACN) and 0.1% TFA, dried in the speed vacuum concentrator, and stored at −80°C prior to mass spectrometry analysis. Purified peptides were labeled with TMT10plex Isobaric Label Reagent Set (Thermo Fisher Scientific, USA) according to the manufacturer’s protocol with some modifications. Twenty-five micrograms of peptide mixture extracted from each sample was labeled with different isobaric tags. For labeling, 41 µL of different TMT Isobaric Label Reagents was added to 90 µL of each sample containing the purified peptides. Reaction mixtures were incubated for an hour followed by the addition of 9 µL of 1 M Tris-HCl, pH 8.0 to quench the labeling reaction and incubated for 15 min at RT. Thirty microliters of 10% TFA was added to the samples to acidify the solution. The labeled peptides were purified on C18 SepPack columns (Teknokroma) and eluted with 800 µL of 60% ACN and 0.1% TFA, dried in a speed vacuum concentrator, and stored at −80°C prior to mass spectrometry. Mass-spectrometry measurements were performed on a Q Exactive HF coupled to the ACQUITY UPLC M-Class System (Waters). A total of 5 µL of the samples was loaded onto an ACQUITY UPLC M-Class Peptide CSH column [75 µm inner diameter, 25 cm length, and 1.7 µm bead size (Waters)] at a flow rate of 0.4 µL min^−1^ in a 0.1% (vol/vol) formic acid solution. Peptide elution was performed by increasing the acetonitrile gradient from 0% to 12% (vol/vol) over 20 min, from 12% to 24% for the next 70 min, from 24% to 36% for 30 min, and from 36% to 85% for the last minute. The column was then washed with 85% (vol/vol) acetonitrile for 5 min, at a flow rate of 0.3 µL/min. Peptide ions were detected in a full scan from a mass-to-charge ratio of 300 to 1,600 at a resolution of 120,000. tandem mass spectrometry (MS/MS) scans were performed for the 10 peptides with the highest MS signal (ddMS2 resolution of 15,000, AGC target 10^5^, isolation width mass-to-charge ratio 1.2 *m*/*z*, and relative collision energy 27). Peptides for which MS/MS spectra had been recorded were excluded from further MS/MS scans for 30 seconds. Quantitative analysis of MS/MS measurements was performed with MaxQuant software (77); the results of the different protein abundances are listed in Tables S2 to S5. *E. coli* protein sequences were used by the search engine Andromeda for the identification of peptides. The settings used for the search: 10 ppm peptide mass tolerance; 0.8 Da MS/MS tolerance; a maximum of two missed cleavages allowed; the threshold for validation of peptides set to 0.01 using a decoy database; carboxamidomethylation of cysteine was set as a fixed modification; and oxidation of methionine was set as a variable modification. The minimum peptide length of six amino acids was used. The quantification was performed for proteins with a minimum of one unique and one razor peptide. Known contaminants, such as keratins, were removed from further analysis.

### Construction of the *narG-lacZ, iscR-lacZ, and moeA-lacZ* fusions

The promoter fragment of *narGHJI* for *lacZ* fusion was synthesized by Thermo Fischer Scientific, USA and was cloned into *Sma*I and *Bam*HI sites of the pGE593 vector containing the *lacZ* gene downstream of the multiple cloning site to create *narG-lacZ*. For the *iscR-lacZ* plasmid, the gene region from −200 to −1 bp upstream of the *iscR* transcriptional start site was cloned into the *EcoR*I and *Bam*HI sites of the pGE593 vector. The *moeA-lacZ* fusion was constructed using promoter region from −200 bp until ATG of *moeA*, which was cloned into the *Eco*RI and *Bam*HI sites of the pEG593 vector.

### Quantification of β-galactosidase activities

To study the regulation of the *torCAD* operon, the *torC-lacZ* fusion from the plasmid pPTor3 was used, which contains the 143-bp *torC* promoter region together with 276 bp of the *torC* gene fused to the *lacZ* gene (63).

Cells were grown anaerobically in the presence of 20 mM TMAO or 20 mM KNO_3_ and 0.1% glycerol at 37°C until the mid-log phase (OD_600 nm_ = 0.5–1.5), and β-galactosidase activities were measured by using the SDS-chloroform method. Bacterial cells in 500 µL of Z buffer (60 mM Na_2_HPO_4_, 40 mM NaH_2_PO_4_, 10 mM KCl, 1 mM MgSO_4_, and 0.05 mM β-mercaptoethanol, pH 8.0) were permeabilized with 25 µL of 0.1% SDS and 50 µL of chloroform. Samples were incubated at 28°C for 5 min. Reactions were started by the addition of 100 µL of o-nitrophenyl-β-D-galactoside in a concentration of 4 mg/mL. Reactions were stopped by the addition of 0.25 mL of 1 M Na_2_CO_3_. The amount of formed *o*-nitrophenol was measured at 420 nm, corrected for light scattering at 550 nm, and normalized to the volume of cells, their optical density at 600 nm, and the reaction time (Miller units). For each assay, a respective blank reaction containing BW25113 cells transformed with the empty pGE593 vector control was subtracted.

### TMAO reductase activity

Fifty milliliters of bacterial cells was grown anaerobically in the presence of 20 mM TMAO and 0.1% glycerol. When cells reached the mid-log phase (4 h), cells were pelleted and resuspended in 15 mL of 100 mM phosphate buffer, pH 6.5 (100 mM K_2_HPO_4_ and 100 mM KH_2_PO_4_). Cells were lysed by sonification. A total of 500 µL of cell extract was incubated under anaerobic conditions for 3 h at 4°C. TMAO reductase activity of 30 µL lysate was recorded after the addition of 40 µL 15 mM TMAO, 80 µL Tris-HCL pH 6.8, 80 µL 15 mM benzyl viologen, and 3,770 µL 100 mM phosphate buffer containing sodium dithionite. The activity was calculated using the equation: U (mol/min)/mL = 0.5× (ΔAbs_600 nm_/min)/ε_benzyl viologen_/V_lysate_.

### Detection of Moco in cell extracts

Bacteria were grown anaerobically at 37°C with 20 mM TMAO and 0.1% glycerol until the cultures reached the mid-log phase, followed by centrifugation, and sonicated in 100 mM Tris-HCl, pH 7.2. Moco was quantified by adding 50 µL of solution A (1,063 µL of KI solution and 100 µL of 37% HCl; KI solution was prepared by dissolving 1 g of I_2_ and 2 g of KI in 91.4 mL of Millipore water) and 150 µL of KI solution to 400 µL of bacterial lysate. Samples were incubated at 95°C for 30 min and kept in the dark at RT overnight. After centrifugation, 100 µL of 1% ascorbic acid and 100 µL of 1 M Tris-HCl were added. FormA was obtained after the addition of 40 mM MgCl_2_ and 1 U of fast alkaline phosphatase. For purification of FormA from the extract, the samples were loaded onto a 500 µL QAE ion exchange resin (Sigma) equilibrated in water. The column was washed with 10 column volumes of water and with 1,300 µL of 10 mM acetic acid. FormA was eluted six times with 500 µL of 10 mM acetic acid. The fractions were separated on a C-18 reverse-phase HPLC column (4.6 × 250 mm ODS Hypersil; particle size, 5 µm) equilibrated in 5 mM ammonium acetate, 15% (vol/vol) methanol at a flow rate of 1 mL/min. Elution of FormA was monitored by an Agilent 1100 series fluorescence detector using *λ*_ex_ = 383 nm and *λ*_em_ *=* 450 nm. The total FormA content was normalized to the OD_600 nm_.

### Protein expression and purification

#### ArcA

For overexpression of the ArcA, 1 L LB medium containing *E. coli* BL21 (DE3) cells transformed with plasmid pK9431 (47) were grown at 37°C until OD_600 nm_ = 0.5 was reached. One millimolar of IPTG was added, and growth at 30°C was continued for an additional 6 h that was followed by centrifugation. The ArcA protein was purified using Ni-NTA standard procedures. The eluted ArcA was incubated overnight with Tobacco Etch Virus proteases at 4°C and was further passed over a Ni^2+^-agarose column to remove His_6_-Tag.

#### TorR

For overexpression of the TorR, 100 mL of LB medium containing *E. coli* LCB620 strain transformed with plasmid pGS1 (1) was grown at 37°C until OD_600 nm_ = 0.5 was reached. One millimolar of IPTG was added, and growth at 37°C was continued for further 1 h followed by centrifugation. The TorR protein was purified using 1 mL heparin sepharose column, eluted with concentration-dependent KCl containing 40 mM Tris-HCl, pH 7.6 as described previously (1).

#### FNR

For the overexpression of FNR protein, 2 L of LB-tryptone media containing *E. coli* BL21 (DE3) strain transformed with *pMW68* plasmid was grown at 37°C for 2 h. Seventy micromolars of IPTG was added, and it was grown at 37°C for an hour. About 200 µM ammonium iron (III) was added into the culture and incubated at 37°C overnight under anaerobic conditions followed by centrifugation. For the purification of anaerobic FNR, the cells were sonicated and the supernatant from the crude extract was passed through a glutathione–Sepharose 4B column. The elution was performed with 50 mm Tris-HCl, pH 7.6 buffer, and 40 U thrombin was applied for 3 h, as described previously (78).

### Quantification of total porphyrin content

The porphyrin quantification method was adapted as previously described with some modifications (2, 4). The cells were cultivated with 20 mM TMAO under anaerobic conditions in 50 mL falcon tubes. The cells were harvested after 8 h of cultivation and were resuspended in 50 mM Tris-HCl, pH 8.0 for sonication. After sonication, 200 µL of crude extract was mixed with 400 µL of ethyl acetate/acetic acid (4:1, vol/vol) and centrifuged for 5 min at room temperature. After centrifugation, the organic phase was transferred into a new tube and mixed with 400 µL of 1.5 N HCl. Finally, the aqueous phase was measured in fluorescence spectroscopy at *λ*_ex_ = 409 nm and *λ*_em_ = 600 nm emission. The organic phase was reextracted by fresh 1.5 N HCl until no fluorescence remained detectable.

### proFIRE for protein-DNA interactions

proFIRE system facilitates the purification of pure homogenous protein-DNA conjugates, using an equilibration buffer (50 mM Na_2_HPO_4_/NaH_2_PO_4_ pH 7.2 and 150 mM NaCl) and an elution buffer (50 mM Na_2_HPO_4_/NaH_2_PO_4_ pH 7.2 and 1 M NaCl). After the injection of the conjugate, the system algorithm selects the required program based on the DNA length and assesses three aspects: (i) protein purity, (ii) protein-DNA conjugate, and (iii) free DNA, as three peaks in the chromatogram. In our experiment, the phosphorylated ArcA and TorR proteins interact with the *moeAB* and *torCAD* operon under anaerobic conditions. Although proFIRE usually operates under aerobic conditions, to make the purification possible anaerobically, the system was calibrated with anaerobic buffers and the sample preparation was subsequently performed anaerobically. The preparation was carried out in three steps: (i) phosphorylation of proteins: the binding of the regulatory proteins TorR and ArcA to their respective operon sites solely depends on the phosphorylation of respective proteins under anaerobic conditions and site-specific binding of the proteins. A total of 100 µg of TorR and ArcA was incubated with 50 mM disodium carbamoyl phosphate in 50 mM Tris, 150 mM NaCl, 1 mM MgCl_2_, pH 7.9 for 1 h at 30°C under anaerobic conditions. (ii) protein-DNA conjugation: the phosphorylated proteins were incubated with 500 nM of the respective operon DNA. The incubation was performed for the phosphorylated ArcA-*torCAD*, ArcA-*moeAB*, and TorR-*torCAD* at room temperature for 30 min under anaerobic conditions. (iii) injection into proFIRE system: the conjugate volume was made up to 160 µL using anaerobic 50 mM Na_2_HPO_4_/NaH_2_PO_4_ pH 8.0 and 150 mM NaCl buffer and injected via an injection syringe into the proFIRE. The DBS-chromatographic column was purchased from Dynamic Biosensors GmBH, compatible with the proFIRE system. The flow rate was maintained at 1 mL/min.

## SUPPLEMENTAL MATERIAL

Supplemental Material spectrum.03480-23-s0001.pdf Supplemental figures and tables.

ASM does not own the copyrights to Supplemental Material that may be linked to, or accessed through, an article. The authors have granted ASM a non-exclusive, world-wide license to publish the Supplemental Material files. Please contact the corresponding author directly for reuse.
