# Supplementary material for: Iron limitation indirectly reduces the Escherichia coli torCAD operon expression by a reduction of molybdenum cofactor availability
Source: Microbiol Spectr. 2024 Jan 9;12(2):e03480-23. doi: 10.1128/spectrum.03480-23 (PMC10845959; doi:10.1128/spectrum.03480-23)
Supplement: Supplemental Material — Supplemental figures and tables. [file spectrum.03480-23-s0001.pdf]

Supplementary material

Iron limitation indirectly reduces the *Escherichia coli torCAD* operon expression by a reduction of molybdenum cofactor availability

Muhammad Abrar Hasnat[1], Arkadiusz Zupok[1], Michal Gorka[2], Chantal Iobbi-Nivol[3], Aleksandra Skirycz[2], Cécile Jourlin-Castelli[3], Frank Bier[4], SaloniAgarwal[4], Ehizode Irefo[1] and Silke Leimkühler[1,*]

[1] University of Potsdam, Institute of Biochemistry and Biology, Department of Molecular Enzymology, Karl-Liebknecht Str. 24-25, 14476, Potsdam-Golm, Germany

[2] Max-Planck-Institute of Molecular Plant Physiology, Am Mühlenberg 2, 14476, Potsdam-Golm, Germany

[3] Aix-Marseille Université, Centre National de la Recherche Scientifique, Institut de Microbiologie de la Méditerranée, Laboratoire de Bioénergétique et Ingénierie des Protéines, Marseille, France

[4] University of Potsdam, Institute of Biochemistry and Biology, Department of Molecular Analytic and electronic, Karl-Liebknecht Str. 24-25, 14476, Potsdam-Golm, Germany

Running title: The regulation of *torCAD* operon expression

Supplementary Tables:

Table S1:Strains and plasmids used in this study

Plasmid or strain	Genotype or relevant characteristics	Reference
Ptor3	Gene region -276 bp to -1 bp upstream of *torC* transcriptional start cloned into SmaI site of pGE593, Amp^r	(1)
Ptor40	Gene region -87 bp to -22 bp upstream of *torC* and gene region -18 to +15 relative to *narG* transcription start site (Hybrid promoter of *torC* and *narG*) transcriptional start cloned into SmaI site of pGE593, Amp^r	(2)
Ptor43	Gene region -86 bp to +1 bp upstream of *torC* transcriptional start cloned into SmaI site of pGE593, Amp^r	(2)
Ptor52	Gene region -142 bp to +31 bp upstream of *torC* transcriptional start cloned into EcoRI/BamHI site of pGE593, Amp^r	This study
Ptor7	Gene region -124 bp to +48 bp upstream of *torC* transcriptional start cloned into SmaI site of pGE593, Amp^r	(2)
Ptor5	Gene region upstream of torC from *Salmonella Typhimurium*	(2)
piscR-lacZ	Gene region 200 bp to 21 bp upstream of *iscR* transcriptional start cloned into EcoRI/BamHI sites of pGE593, Amp^r	(3)
pfnr-lacZ	Gene region 200 bp to 21 bp upstream of *fnr* transcriptional start cloned into EcoRI/BamHI site of pGE593, Amp^r	This study
pmoeA-lacZ	Gene region 200 bp to 200 bp upstream of *moeA* transcriptional start cloned into EcoRI/BamHI site of pGE593, Amp^r	This study
pnarGHJI-lacZ	Gene region from the promoter fragment of narGHJI cloned into SmaI/BamHI site of pGE593, Amp^r	This study
piscSUAHscABX	*iscSUAHscABX* coding region cloned into EcoRI/XhoI of *pCDF-duet1*, $Spec^r$	This study
pK9431	*arcA* coding region cloned into NheI/XhoI of *pET21d*, Amp^r	(4)
pMW68	*fnr* coding region cloned into BamHI/SmaI of *pGEX-4T-1*, Amp^r	(5)
pGS1	*torR* coding region cloned into EcoRI/BamHI of pJF119EH, Amp^r	(6)
pPH151	*iscRSUAHscABX* coding region cloned	(7)
BW25113	*laclq rrnBT14 lacZWJ16 hsdR514 araBADAH33 rhaBADLD78*	
Δ*nac* strain	BW25113 derivate, Δ*nac::kan*	(8)
Δ*arcA* strain	BW25113 derivate, Δ*arcA::kan*	(8)
Δ*ccmA* strain	BW25113 derivate, Δ*ccmA::kan*	(8)
Δ*fnr* strain	BW25113 derivate, Δ*fnr::kan*	(8)
Δ*fur* strain	BW25113 derivate, Δ*fur::kan*	(8)
Δ*torI* strain	BW25113 derivate, Δ*torI::kan*	(8)
Δ*iscR* strain	BW25113 derivate, Δ*iscR::kan*	(8)
Δ*iscU* strain	BW25113 derivate, Δ*iscU::kan*	(8)

Δ*narL* strain	BW25113 derivate, Δ*narL::kan*	(8)
Δ*modE* strain	BW25113 derivate, Δ*modE::kan*	(8)
Δ*moaA* strain	BW25113 derivate, Δ*moaA::kan*	(8)
Δ*moaB* strain	BW25113 derivate, Δ*moaB::kan*	(8)
Δ*moaC* strain	BW25113 derivate, Δ*moaC::kan*	(8)
Δ*moaD* strain	BW25113 derivate, Δ*moaD::kan*	(8)
Δ*moaE* strain	BW25113 derivate, Δ*moaE::kan*	(8)
Δ*moeA* strain	BW25113 derivate, Δ*moeA::kan*	(8)
Δ*moeB* strain	BW25113 derivate, Δ*moeB::kan*	(8)
Δ*mogA* strain	BW25113 derivate, Δ*mogA::kan*	(8)
Δ*mobA* strain	BW25113 derivate, Δ*mobA::kan*	(8)
Δ*mocA* strain	BW25113 derivate, Δ*mocA::kan*	(8)
RK4353 strain	DE (argF-lac)169 DE(fimB-fimE)632(::IS1) DE(fruK-yeiR)725(fruA25) F- Lambda- [araD139]B/r deoC1 e14- flhD5301 gyrA219 non-9 rbsR22 relA1 rpsL150(strR)	(9)
RK5200 strain	RK4353 derivate, Δ*moaA*	(9)

Table S2: Proteomic data higher abundance in LB against LB + 150 µM DIP

The detectable proteins that identified by proteomics analysis with and without 150 mM 2,2-DIP. The listed protein names with corresponding fold changes in the presence of 150 mM 2,2-DIP compared to without 150 mM 2,2-DIP. The strain was cultivated for 4 h in LB media at 37 °C. Green arrow shows increased fold.

Protein name	Only LB	LB+150 DIP (Fold change)
6PGD	1	↑ 1.182
DHE4	1	↑ 1.555
DHNA	1	↑ 2.158
SODM	1	↑ 2.805
RIR1	1	↑ 1.911
AAT	1	↑ 1.159
PTM3C	1	↑ 1.291
RPOD	1	↑ 1.010
DPO1	1	↑ 1.140
DCDA	1	↑ 2.137
CAPP	1	↑ 1.010
AROH	1	↑ 1.592
SDHD	1	↑ 1.287
MANA	1	↑ 1.315
SYW	1	↑ 1.066
SYI	1	↑ 1.049
SYA	1	↑ 1.012
SYM	1	↑ 1.032
SYGA	1	↑ 1.032
SYGB	1	↑ 1.112
SYQ	1	↑ 1.012
MALF	1	↑ 1.214
PBPB	1	↑ 1.129
RBSB	1	↑ 1.184
LAMB	1	↑ 1.200
UVRD	1	↑ 1.044
MIOC	1	↑ 1.119
MALM	1	↑ 1.028
DAPB	1	↑ 1.049
GUAA	1	↑ 1.484
FRSA	1	↑ 1.618
GSHB	1	↑ 1.151
TYRB	1	↑ 1.388
SYE	1	↑ 1.203
AMPN	1	↑ 1.203
RBSD	1	↑ 1.159
EX7L	1	↑ 1.464
PYRC	1	↑ 1.153
AROD	1	↑ 1.334
PDXB	1	↑ 1.077
APAH	1	↑ 1.088
SRLD	1	↑ 1.135
ILVC	1	↑ 1.163
BGLR	1	↑ 1.480
FEPA	1	↑ 130.004
TSAD	1	↑ 1.351
DLD	1	↑ 1.535
BTUE	1	↑ 1.282
ERA	1	↑ 1.113
GSHR	1	↑ 1.555
METC	1	↑ 1.372
ODP2	1	↑ 1.069
FHUA	1	↑ 42.031
HEM3	1	↑ 1.323
HIS8	1	↑ 1.268
HIS7	1	↑ 1.789
HISX	1	↑ 1.104
HIS2	1	↑ 1.551
RSMA	1	↑ 1.279
PFKB	1	↑ 2.186
POXB	1	↑ 1.605
PROA	1	↑ 1.014
RF2	1	↑ 1.055

NAGB	1	↑	1.219
ORN	1	↑	1.310
PYRB	1	↑	1.005
PAND	1	↑	1.105
PDXJ	1	↑	1.215
PFKA	1	↑	1.503
PGK	1	↑	1.313
PIMT	1	↑	1.122
PLSB	1	↑	1.119
PPK1	1	↑	1.296
NADK	1	↑	1.362
LEXA	1	↑	1.162
PTH	1	↑	1.709
PURA	1	↑	1.101
PYRH	1	↑	1.764
SPED	1	↑	1.867
QUEA	1	↑	1.243
RECA	1	↑	1.177
RIBA	1	↑	1.243
RL34	1	↑	1.106
RNPA	1	↑	1.086
RPIA	1	↑	1.459
RUVB	1	↑	1.657
SSRP	1	↑	1.224
SUCC	1	↑	1.476
SURE	1	↑	1.380
TOLB	1	↑	1.211
TPIS	1	↑	1.048
TPX	1	↑	2.486
TALA	1	↑	1.571
TALB	1	↑	1.109
TRMD	1	↑	1.002
TRPA	1	↑	2.375
TRPB	1	↑	1.151
RAPZ	1	↑	1.515
YFCN	1	↑	1.099
YBJQ	1	↑	1.136
YGFB	1	↑	1.088
NRDR	1	↑	1.145
YMDB	1	↑	1.910
YACL	1	↑	1.552
YAJQ	1	↑	1.164
UPP	1	↑	1.042
UVRB	1	↑	1.530
EX7S	1	↑	1.423
YIHI	1	↑	1.256
YAAA	1	↑	1.533
TRMB	1	↑	1.163
RLMH	1	↑	1.122
YBED	1	↑	1.343
RHLB	1	↑	1.032
PSD	1	↑	1.296
YAEP	1	↑	2.073
YEEX	1	↑	1.800
MATP	1	↑	1.392
SYK2	1	↑	1.040
FETP	1	↑	1.028
RRAA	1	↑	1.123
PRMA	1	↑	1.711
SYDP	1	↑	1.037
SLYA	1	↑	1.100
YQGE	1	↑	1.192
YFBU	1	↑	1.653
YJGA	1	↑	1.228
YIDA	1	↑	1.313
YCIA	1	↑	1.654
BAMC	1	↑	1.169
OMPA	1	↑	1.052
OMPX	1	↑	1.577

SDHB	1	⇧	1.815
PPA	1	⇧	1.137
TYRR	1	⇧	1.287
RECC	1	⇧	1.766
DDLB	1	⇧	1.434
TDH	1	⇧	2.514
FOLC	1	⇧	1.011
NIRB	1	⇧	1.594
PYRF	1	⇧	1.640
MURB	1	⇧	1.184
USG	1	⇧	1.632
SPPA	1	⇧	1.524
DNAJ	1	⇧	1.101
AK3	1	⇧	1.560
PTFX1	1	⇧	1.556
MASY	1	⇧	1.045
EX3	1	⇧	1.255
AVTA	1	⇧	1.519
GAL7	1	⇧	1.058
SPEE	1	⇧	1.047
ASNB	1	⇧	1.090
PFLB	1	⇧	1.508
MTLD	1	⇧	1.399
PUTA	1	⇧	2.065
UVRA	1	⇧	1.258
ALR1	1	⇧	1.030
END4	1	⇧	1.470
AROA	1	⇧	1.258
ASSY	1	⇧	1.269
ATPE	1	⇧	1.047
BIOD2	1	⇧	1.699
CARA	1	⇧	1.187
GLPK	1	⇧	1.158
CH60	1	⇧	1.254
CH10	1	⇧	1.460
PNCC	1	⇧	2.270
HSLU	1	⇧	1.005
CLSA	1	⇧	1.157
COAD	1	⇧	2.120
DADA	1	⇧	1.231
DAPF	1	⇧	1.559
DEOB	1	⇧	2.932
DEOC	1	⇧	1.098
DAPA	1	⇧	1.061
HSCB	1	⇧	1.799
DSBB	1	⇧	2.446
DER	1	⇧	1.119
ENGB	1	⇧	1.651
ENO	1	⇧	1.172
FABZ	1	⇧	1.482
G6PI	1	⇧	1.330
GCH1	1	⇧	1.191
GLGA	1	⇧	1.088
GLK	1	⇧	1.172
GSH1	1	⇧	1.180
HFQ	1	⇧	1.244
IHFB	1	⇧	1.169
HSLO	1	⇧	1.294
DNAK	1	⇧	1.319
HTPG	1	⇧	1.248
KDSA	1	⇧	1.083
KPRS	1	⇧	1.051
KTHY	1	⇧	1.113
LPXA	1	⇧	1.110
LPXC	1	⇧	2.160
NTPPB	1	⇧	1.255
MSCL	1	⇧	1.220
MSRA	1	⇧	1.033
MSRB	1	⇧	1.020

MLTA	1	⇧	1.196
BAME	1	⇧	1.054
ALKH	1	⇧	1.071
ALAA	1	⇧	1.440
ASPG1	1	⇧	1.187
ALF1	1	⇧	1.565
FUR	1	⇧	1.187
G3P1	1	⇧	1.426
GLPA	1	⇧	1.552
GSTA	1	⇧	1.649
CYSE	1	⇧	1.232
DAPD	1	⇧	1.145
CYSB	1	⇧	1.202
ACEA	1	⇧	1.478
MUG	1	⇧	1.476
LDCI	1	⇧	1.800
GCVR	1	⇧	2.098
NIRD	1	⇧	1.539
CMPDT	1	⇧	1.642
PHOL	1	⇧	1.030
PHOU	1	⇧	1.361
SLYD	1	⇧	1.343
PPIC	1	⇧	1.185
P5CR	1	⇧	1.050
LON	1	⇧	1.238
XGPT	1	⇧	1.134
TRXB	1	⇧	1.266
ADHE	1	⇧	2.047
DHAS	1	⇧	1.286
TAS	1	⇧	1.642
YBIT	1	⇧	1.067
IBAG	1	⇧	1.011
YRDA	1	⇧	1.247
MREB	1	⇧	1.071
PTHP	1	⇧	1.251
OMPR	1	⇧	1.062
THIO	1	⇧	1.284
RLUC	1	⇧	1.029
RSUA	1	⇧	1.253
YEAY	1	⇧	1.436
UBID	1	⇧	1.283
GALF	1	⇧	1.076
USPD	1	⇧	1.945
USPE	1	⇧	1.306
CADB	1	⇧	1.788
ARTP	1	⇧	1.489
PSTB	1	⇧	1.624
SAPD	1	⇧	1.136
FTSH	1	⇧	1.094
FABD	1	⇧	1.236
FRMR	1	⇧	1.116
YBCJ	1	⇧	1.285
YBEL	1	⇧	1.386
YBIS	1	⇧	1.466
YCCF	1	⇧	1.080
EFEO	1	⇧	3.532
YCEB	1	⇧	2.064
YCGL	1	⇧	1.165
YMGD	1	⇧	1.018
YCHN	1	⇧	1.679
YCII	1	⇧	1.651
LAPB	1	⇧	2.199
YCIN	1	⇧	1.279
ALF	1	⇧	1.411
KBL	1	⇧	1.189
ILVE	1	⇧	1.236
PUR8	1	⇧	1.072
AROG	1	⇧	1.396
ATP6	1	⇧	1.235

ATPD	1	↑	1.189
HFLC	1	↑	1.218
HFLK	1	↑	1.101
BCCP	1	↑	1.109
BOLA	1	↑	1.034
CDD	1	↑	1.768
CISY	1	↑	1.359
CYOB	1	↑	2.521
CYOA	1	↑	1.260
CYDA	1	↑	1.104
CYDB	1	↑	1.099
CYSK	1	↑	1.972
DEOD	1	↑	1.585
DYR	1	↑	1.192
DNAG	1	↑	1.165
EXBB	1	↑	27.537
TOLQ	1	↑	1.107
FLAW	1	↑	1.377
KDSC	1	↑	1.238
SURA	1	↑	1.261
DHPS	1	↑	1.588
FOLX	1	↑	1.382
SDHA	1	↑	1.313
G6PD	1	↑	1.399
GLRX2	1	↑	1.617
GLRX3	1	↑	1.168
KDTA	1	↑	1.525
GLO2	1	↑	1.167
PHSG	1	↑	1.147
GNSA	1	↑	1.774
YIBF	1	↑	3.867
SSPA	1	↑	1.110
GSTB	1	↑	1.498
HEM2	1	↑	1.302
HEMG	1	↑	1.169
HEMY	1	↑	1.044
HINT	1	↑	1.201
ROB	1	↑	1.004
LRP	1	↑	1.481
EXUR	1	↑	1.233
ALLR	1	↑	1.445
PURR	1	↑	1.092
OXYR	1	↑	2.259
YEIE	1	↑	1.530
YJDC	1	↑	1.257
LPXL	1	↑	2.178
YDHR	1	↑	1.291
YDJA	1	↑	1.173
YEAG	1	↑	1.657
CUSR	1	↑	1.092
YECA	1	↑	1.237
YEEZ	1	↑	1.167
YEJL	1	↑	1.234
YFCZ	1	↑	1.339
ISPB	1	↑	2.318
IVY	1	↑	1.501
KPYK1	1	↑	1.451
FTSI	1	↑	2.004
NLPD	1	↑	1.207
OSMB	1	↑	2.315
OSME	1	↑	1.110
ECNB	1	↑	1.999
LPTE	1	↑	1.418
LOLC	1	↑	1.116
LPTG	1	↑	1.313
KBP	1	↑	1.669
YGFZ	1	↑	1.260
EDD	1	↑	1.274
IMDH	1	↑	1.783

YECD	1	↑	2.286
YIFL	1	↑	1.056
YIGB	1	↑	1.300
YIHD	1	↑	1.455
YIID	1	↑	1.856
YGAM	1	↑	1.125
RLMM	1	↑	1.243
PPNN	1	↑	1.127
YGGE	1	↑	1.246
YGGN	1	↑	1.395
YGIB	1	↑	1.058
YGIC	1	↑	1.442
YGIM	1	↑	1.359
YGIN	1	↑	1.430
YGIW	1	↑	1.399
LPTA	1	↑	1.010
YHBW	1	↑	2.049
MLAC	1	↑	1.005
YHFA	1	↑	1.144
YHHA	1	↑	2.001
RL16	1	↑	1.067
TRMJ	1	↑	1.182
AHPC	1	↑	1.159
AMN	1	↑	1.208
MAP1	1	↑	1.772
YPDB	1	↑	1.869
YTFL	1	↑	1.189
BCP	1	↑	1.174
CPXR	1	↑	1.279
CREA	1	↑	1.367
CYSG	1	↑	1.477
DACA	1	↑	1.001
YOAB	1	↑	1.183
ARCB	1	↑	1.028
USPA	1	↑	1.121
DCRB	1	↑	1.082
DNAC	1	↑	1.009
DSBA	1	↑	1.540
ELAB	1	↑	1.066
EUTB	1	↑	1.199
FABI	1	↑	1.042
FEOA	1	↑	17.284
FRE	1	↑	1.140
FUCM	1	↑	1.215
GLCG	1	↑	1.171
GLNH	1	↑	1.286
GLPF	1	↑	1.023
GSP	1	↑	2.082
GYRA	1	↑	1.051
HDEA	1	↑	1.732
HDEB	1	↑	1.278
HDED	1	↑	1.567
HDHA	1	↑	1.334
HISJ	1	↑	1.750
SKP	1	↑	1.086
CPDA	1	↑	1.379
K1PF	1	↑	2.419
MALE	1	↑	1.184
MDAB	1	↑	1.688
MIND	1	↑	1.023
MTNN	1	↑	2.145
NAGA	1	↑	1.814
NAGD	1	↑	1.181
YJBR	1	↑	1.446
YJEI	1	↑	1.480
RRAB	1	↑	1.068
RIDA	1	↑	1.313
TABA	1	↑	1.639
NHAB	1	↑	1.432

NUON	1	↑	3.135
NUSA	1	↑	1.065
NUSG	1	↑	1.084
ODO1	1	↑	1.045
ODO2	1	↑	1.107
ODP1	1	↑	1.182
OPPB	1	↑	1.118
OPPC	1	↑	1.055
OSMY	1	↑	1.431
PARC	1	↑	1.121
PDXH	1	↑	1.291
PITA	1	↑	1.013
PMBA	1	↑	1.251
POTD	1	↑	1.197
PPIA	1	↑	1.095
PPX	1	↑	1.041
PROX	1	↑	1.225
PSIF	1	↑	1.972
PSPA	1	↑	1.390
YBBO	1	↑	1.095
GCH1L	1	↑	1.333
YCFH	1	↑	1.630
YCIO	1	↑	1.107
GLRR	1	↑	1.221
RISA	1	↑	1.145
RFAH	1	↑	1.204
RMF	1	↑	2.335
ROF	1	↑	1.095
HPF	1	↑	1.992
RSD	1	↑	2.499
RSEB	1	↑	1.154
SEQA	1	↑	1.266
SSPB	1	↑	1.241
RPE	1	↑	1.070
SOHB	1	↑	1.318
RELA	1	↑	1.106
SPOT	1	↑	1.153
PSTS	1	↑	1.041
YGHA	1	↑	1.135
SECF	1	↑	1.298
RPOE	1	↑	1.013
SLT	1	↑	1.302
SODC	1	↑	1.452
SRP54	1	↑	1.136
SSB	1	↑	1.214
CHRR	1	↑	1.789
SUCD	1	↑	1.243
THIL	1	↑	1.291
TESB	1	↑	1.019
THIO2	1	↑	1.398
TLDD	1	↑	1.171
RBSC	1	↑	1.077
TRKA	1	↑	1.794
YFIF	1	↑	1.078
SYY	1	↑	1.200
YHBY	1	↑	1.033
PPNP	1	↑	1.579
OSMC	1	↑	1.710
MSCS	1	↑	2.447
DEGP	1	↑	1.679
RCSB	1	↑	1.054
GLNQ	1	↑	1.300
HIS4	1	↑	1.928
ENTE	1	↑	22.174
NARU	1	↑	2.193
NARV	1	↑	1.699
ENTF	1	↑	22.168
UNG	1	↑	1.196
UDP	1	↑	1.482

YBHB	1	↑	2.323
BIOH	1	↑	1.059
GLPB	1	↑	1.815
GLPD	1	↑	4.328
FECA	1	↑	2898.357
TREA	1	↑	1.295
SELB	1	↑	1.238
TOP3	1	↑	1.005
MURD	1	↑	1.276
FECB	1	↑	31.880
DNLJ	1	↑	1.223
GLGX	1	↑	1.468
PUR4	1	↑	1.008
AROE	1	↑	1.161
SDHL	1	↑	1.427
MIAA	1	↑	1.641
SELD	1	↑	1.166
SYP	1	↑	1.203
YJDN	1	↑	2.524
CYSP	1	↑	1.414
FHUE	1	↑	3.598
MREC	1	↑	1.266
NFSA	1	↑	1.932
BETB	1	↑	1.880
MURC	1	↑	1.150
UBIG	1	↑	1.282
MINC	1	↑	1.259
NADE	1	↑	1.598
AGP	1	↑	1.030
PTFBC	1	↑	1.772
SPEA	1	↑	1.065
CATE	1	↑	1.108
RNI	1	↑	2.329
YMDF	1	↑	5.656
YCIF	1	↑	1.842
YCIE	1	↑	2.419
YCAC	1	↑	1.612
RNR	1	↑	1.112
SRMB	1	↑	1.168
KPYK2	1	↑	1.000
YBHA	1	↑	1.325
SYD	1	↑	1.096
ASNB	1	↑	1.306
MURE	1	↑	1.100
CYSQ	1	↑	1.578
GABT	1	↑	1.546
ADD	1	↑	1.632
MURI	1	↑	2.217
YIGA	1	↑	1.111
SERC	1	↑	1.462
PSS	1	↑	1.172
PHOP	1	↑	1.013
YICC	1	↑	1.088
OPPA	1	↑	1.128
DPPA	1	↑	1.796
PRC	1	↑	1.334
PPIB	1	↑	1.003
GSA	1	↑	1.236
HTPX	1	↑	1.282
ARGE	1	↑	1.823
MUTS	1	↑	1.986
MAK	1	↑	1.003
DCP	1	↑	1.344
FOLD	1	↑	1.685
MRR	1	↑	1.192
LPXM	1	↑	1.171
HMP	1	↑	4.146
RP54	1	↑	1.941
TEHB	1	↑	1.383

FRMA	1	↑	1.037
HFLX	1	↑	1.179
MNME	1	↑	1.009
CODA	1	↑	1.535
GABD	1	↑	1.345
UBIH	1	↑	1.805
UBII	1	↑	1.372
RIBD	1	↑	1.117
GPPA	1	↑	1.115
ALDA	1	↑	1.570
METE	1	↑	4.148
MSYB	1	↑	1.436
MNMA	1	↑	1.090
HFLD	1	↑	1.071
RHLE	1	↑	1.044
LOIP	1	↑	1.503
PDXI	1	↑	1.416
APPC	1	↑	1.066
AMY2	1	↑	1.181
MAO1	1	↑	1.182
PLSC	1	↑	1.446
FTSP	1	↑	1.483
WAAU	1	↑	1.071
GLND	1	↑	1.336
AHR	1	↑	1.994
NADR	1	↑	1.391
OPDA	1	↑	1.306
LPXK	1	↑	1.632
ROXA	1	↑	2.407
RODZ	1	↑	1.023
ACSA	1	↑	1.129
WECB	1	↑	1.024
WECE	1	↑	1.363
YIGL	1	↑	2.118
QOR1	1	↑	1.488
ADIA	1	↑	1.761
FENR	1	↑	2.466
YCGB	1	↑	1.244
CYDD	1	↑	1.667
FTSN	1	↑	1.294
YCEH	1	↑	1.158
DCUP	1	↑	1.208
NADC	1	↑	1.083
YBIB	1	↑	1.081
HCXB	1	↑	1.424
RNB	1	↑	1.056
ARTI	1	↑	1.419
DKGB	1	↑	2.869
YAFC	1	↑	2.403
GLNE	1	↑	1.194
3PASE	1	↑	1.116
MFD	1	↑	1.300
PANB	1	↑	1.186
YEDD	1	↑	1.213
KCH	1	↑	1.147
THTM	1	↑	1.230
ACRF	1	↑	1.019
YICH	1	↑	1.580
RAVA	1	↑	1.252
HCHA	1	↑	1.659
PRPC	1	↑	1.088
PANC	1	↑	1.816
OTSA	1	↑	1.815
OTSB	1	↑	1.484
DJLA	1	↑	1.163
NNR	1	↑	1.013
LPLA	1	↑	1.111
HEMN	1	↑	1.394
TYPA	1	↑	1.048

YIIM	1	↑	1.046
YIIS	1	↑	1.021
NUDC	1	↑	1.218
DUSA	1	↑	1.064
SBMC	1	↑	1.701
YEIR	1	↑	1.053
OPGG	1	↑	1.154
YEBE	1	↑	1.540
YEBF	1	↑	1.780
YEHS	1	↑	1.036
YOHF	1	↑	2.537
TKT2	1	↑	1.701
NIKD	1	↑	1.183
NIKE	1	↑	1.288
RLUD	1	↑	1.146
FEOB	1	↑	220.848
NDPA	1	↑	1.558
YDCF	1	↑	1.350
AHPF	1	↑	1.169
AMIA	1	↑	1.195
CUEO	1	↑	1.140
CBPA	1	↑	1.090
ZAPD	1	↑	1.293
RDGC	1	↑	1.021
YADG	1	↑	1.180
YEGD	1	↑	1.005
PURU	1	↑	2.147
PEPB	1	↑	1.270
NUDK	1	↑	1.296
PT1P	1	↑	1.206
SLP	1	↑	2.035
DCTR	1	↑	1.254
YHJA	1	↑	1.826
MASZ	1	↑	1.062
ZNTA	1	↑	1.517
RLMJ	1	↑	1.895
MDTE	1	↑	1.206
MDTF	1	↑	1.457
YHJD	1	↑	1.344
YHJG	1	↑	1.232
YHJJ	1	↑	1.095
GHRB	1	↑	1.240
ALDB	1	↑	1.392
GPMI	1	↑	1.285
RMLC	1	↑	1.587
MPL	1	↑	1.148
GLTI	1	↑	1.475
USPF	1	↑	1.098
NFSB	1	↑	1.259
YGGL	1	↑	1.096
DEGQ	1	↑	1.489
YEAD	1	↑	1.982
ERFK	1	↑	1.099
AG43	1	↑	1.070
ALSB	1	↑	1.013
RSGA	1	↑	1.122
YTFQ	1	↑	1.378
YJGR	1	↑	2.631
YJHU	1	↑	1.036
RSMC	1	↑	1.509
YJJU	1	↑	1.122
NCPP	1	↑	2.469
ADHP	1	↑	2.586
YBAL	1	↑	1.315
RCSD	1	↑	1.084
NLPE	1	↑	1.152
MTOX	1	↑	1.058
MLTB	1	↑	1.060
AZOR	1	↑	1.744

MLAE	1	↑	2.151
YGDI	1	↑	1.978
QUED	1	↑	1.178
BEPA	1	↑	1.197
PLPHP	1	↑	1.015
YFCE	1	↑	1.094
YQFB	1	↑	1.994
HLDD	1	↑	1.240
GRCA	1	↑	2.253
MALG	1	↑	1.612
MALK	1	↑	1.222
YJBJ	1	↑	1.349
AMPA	1	↑	1.070
BAER	1	↑	1.586
YTFE	1	↑	5.374
PTGA	1	↑	1.104
PTGCB	1	↑	1.029
PTND	1	↑	1.279
PTFAH	1	↑	1.936
POTA	1	↑	1.177
DCEB	1	↑	1.680
FUCI	1	↑	1.050
RIR2	1	↑	1.851
YAHK	1	↑	1.567
YAHO	1	↑	1.105
UBIF	1	↑	1.912
YBFF	1	↑	1.153
PXPA	1	↑	1.106
FIU	1	↑	2.835
YBJI	1	↑	2.257
YBJP	1	↑	1.167
YCAO	1	↑	1.245
GLO22	1	↑	1.403
YCBX	1	↑	1.333
RLMKL	1	↑	1.046
LONH	1	↑	1.102
YCCU	1	↑	1.642
GHRA	1	↑	2.189
NAGZ	1	↑	1.341
LOLD	1	↑	1.083
NPD	1	↑	1.330
PLIG	1	↑	1.617
YCGM	1	↑	1.817
LDCA	1	↑	1.094
DHAK	1	↑	1.318
YCIT	1	↑	1.025
CURA	1	↑	1.384
YNCE	1	↑	3155.681
LSRF	1	↑	1.088
YDHF	1	↑	1.131
YNHG	1	↑	1.423
YEAH	1	↑	2.237
YEAO	1	↑	1.332
KDGR	1	↑	1.261
MSRC	1	↑	1.924
RSMF	1	↑	1.053
CMOA	1	↑	1.253
CMOB	1	↑	2.442
DCYD	1	↑	1.574
MPGP	1	↑	2.868
YEGH	1	↑	1.095
YEGP	1	↑	1.191
YEGQ	1	↑	1.403
5DNU	1	↑	1.407
MURQ	1	↑	1.149
YFEY	1	↑	1.147
YFFS	1	↑	1.168
YFGD	1	↑	1.870
YFGM	1	↑	1.103

YGJR	1	↑	1.040
YQJG	1	↑	1.481
HRPA	1	↑	1.276
UUP	1	↑	1.667
KDSD	1	↑	1.031
YHCH	1	↑	1.068
LPOA	1	↑	1.001
YRAR	1	↑	1.115
YHBO	1	↑	2.779
FKBA	1	↑	1.059
DXR	1	↑	1.093
LUXS	1	↑	1.411
YHHW	1	↑	1.776
YHHX	1	↑	1.157
GNTK	1	↑	1.439
YECM	1	↑	1.465
DCLZ	1	↑	1.149
RSTA	1	↑	1.057
LDHD	1	↑	1.448
6PGL	1	↑	1.520
CSIE	1	↑	2.699
YGAP	1	↑	1.825
RAPA	1	↑	1.262
MUKF	1	↑	1.029
RSMH	1	↑	1.093
GUAC	1	↑	1.399
SPEB	1	↑	1.141
MSBA	1	↑	1.104
HIS1	1	↑	1.369
LEPA	1	↑	1.048
SYH	1	↑	1.005
LGT	1	↑	1.125
LOLA	1	↑	1.137
LOLB	1	↑	1.039
CAN	1	↑	2.380
RISB	1	↑	1.000
MDH	1	↑	1.092
FLAV	1	↑	1.249
OPGH	1	↑	1.064
ISPF	1	↑	1.235
GPMA	1	↑	10.994
YAEH	1	↑	1.278
NFUA	1	↑	1.717
RLMB	1	↑	1.295
GMHA	1	↑	1.122
GMHBB	1	↑	1.083
GADC	1	↑	1.489
CBPM	1	↑	1.254
CLPB	1	↑	1.958
YHES	1	↑	1.114
AMIC	1	↑	1.057
YPFJ	1	↑	1.115
YDCL	1	↑	1.502
YDFZ	1	↑	1.407
YDIH	1	↑	1.347
YDIZ	1	↑	1.398
YEAK	1	↑	1.326
YEBV	1	↑	1.620
YEBY	1	↑	1.111
YODD	1	↑	2.091
RCNB	1	↑	2.236
QUEE	1	↑	1.097
SDHE	1	↑	1.280
YGGT	1	↑	1.047
YQJD	1	↑	1.073
YQJE	1	↑	1.330
YQJI	1	↑	1.011
YHBT	1	↑	1.276
MLAD	1	↑	1.033

A2MG	1	↑	1.221
GLAH	1	↑	1.091
HLDE	1	↑	1.023
DSBG	1	↑	1.061
RCLA	1	↑	1.180
PRPD	1	↑	2.622
HXPB	1	↑	1.014
NEMA	1	↑	2.043
FETA	1	↑	1.213
YDGJ	1	↑	1.296
CNOX	1	↑	1.963
YBAT	1	↑	1.595
ALAC	1	↑	1.034
GLSA1	1	↑	1.024
YQAB	1	↑	1.654
PRPB	1	↑	1.328
ASTC	1	↑	1.791
HXPA	1	↑	1.321
ABDH	1	↑	2.192
GNSB	1	↑	1.143
YBAY	1	↑	1.162
THII	1	↑	1.007
YAJO	1	↑	1.303
YNIA	1	↑	1.003
SPY	1	↑	2.406
QUEC	1	↑	1.286
YFCH	1	↑	1.284
CINAL	1	↑	1.034
FSAA	1	↑	2.539
YIBT	1	↑	1.755
BGLA	1	↑	1.162
YGHU	1	↑	1.169
GPR	1	↑	1.020
YQHD	1	↑	2.072
DKGA	1	↑	1.912
UBIK	1	↑	1.218
QUEF	1	↑	1.114
SAPA	1	↑	1.003
YAFV	1	↑	1.278
TRUD	1	↑	1.045
COPA	1	↑	1.838
ADPP	1	↑	1.867

TableS3: Proteomic data lower abundance in LB against LB + 150 µM DIP

The detectable proteins that identified by proteomics analysis with and without 150 mM 2,2-DIP. The listed protein names with corresponding fold changes in the presence of 150 mM 2,2-DIP compared to without 150 mM 2,2-DIP. The strain was cultivated for 4 h in LB media at 37 °C. Red arrow shows reduced fold.

Protein name	Only LB	LB+150 DIP (Fold changes)
INH21	1	↓ 0.637
FRDA	1	↓ 0.202
PHSM	1	↓ 0.794
KHSE	1	↓ 0.455
AK1H	1	↓ 0.279
AK2H	1	↓ 0.950
LEP	1	↓ 0.920
ASPG2	1	↓ 0.219
THRC	1	↓ 0.349
CARB	1	↓ 0.916
RS6	1	↓ 0.737
RS7	1	↓ 0.964
RL15	1	↓ 0.877
ARAF	1	↓ 0.865
TOLC	1	↓ 0.819
PHOE	1	↓ 0.100
DNAA	1	↓ 0.830
FLIC	1	↓ 0.078
KDSB	1	↓ 0.994
RBSA	1	↓ 0.997
EX1	1	↓ 0.947
PNP	1	↓ 0.952
PTRA	1	↓ 0.508
PTHA	1	↓ 0.465
T1SK	1	↓ 0.937
BTUB	1	↓ 0.799
TOP1	1	↓ 0.942
BIRA	1	↓ 0.867
DPO3X	1	↓ 0.716
CCA	1	↓ 0.903
DUT	1	↓ 0.866
FLIM	1	↓ 0.899
MALT	1	↓ 0.608
OMPC	1	↓ 0.828
PNTA	1	↓ 0.884
MCP4	1	↓ 0.018
TYRA	1	↓ 0.509
USHA	1	↓ 0.903
SYV	1	↓ 0.905
SYFB	1	↓ 0.901
AROB	1	↓ 0.995
TYPH	1	↓ 0.942
FDHF	1	↓ 0.195
GLGB	1	↓ 0.950
SYL	1	↓ 0.973
PUR5	1	↓ 0.527
GLPT	1	↓ 0.537
IDH	1	↓ 0.960
SYFA	1	↓ 0.950

CPDB	1	↓	0.710
DACC	1	↓	0.688
T1RK	1	↓	0.768
T1MK	1	↓	0.718
HEMX	1	↓	0.937
GALE	1	↓	0.918
RND	1	↓	0.992
OMPT	1	↓	0.890
PTW3C	1	↓	0.726
GLPQ	1	↓	0.633
GLTB	1	↓	0.235
ACKA	1	↓	0.923
ACP	1	↓	0.891
ARGR	1	↓	0.915
AROK	1	↓	0.804
CLPP	1	↓	0.928
CLPX	1	↓	0.747
KCY	1	↓	0.847
COAA	1	↓	0.708
DDLA	1	↓	0.947
DEF	1	↓	0.922
NANA	1	↓	0.684
UXAB	1	↓	0.573
EFG	1	↓	0.897
EFP	1	↓	0.830
EFPL	1	↓	0.870
EFTS	1	↓	0.880
FABA	1	↓	0.917
FABH	1	↓	0.907
FIS	1	↓	0.665
FLGH	1	↓	0.000
GPDA	1	↓	0.863
GAL1	1	↓	0.614
GCSH	1	↓	0.254
MNMG	1	↓	0.865
GLGC	1	↓	0.765
GREA	1	↓	0.861
IHFA	1	↓	0.933
HSCA	1	↓	0.948
NIKR	1	↓	0.966
IF2	1	↓	0.835
IF3	1	↓	0.785
MGSA	1	↓	0.895
MINE	1	↓	0.967
MURA	1	↓	0.813
NANE	1	↓	0.633
NDK	1	↓	0.994
NUSB	1	↓	0.933
GPMB	1	↓	0.856
IPYR	1	↓	0.999
PROB	1	↓	0.881
HSLV	1	↓	0.780
PEPE	1	↓	0.516
PUR7	1	↓	0.743

PYRD	1	↓	0.882
PYRG	1	↓	0.892
RBFA	1	↓	0.806
RF1	1	↓	0.968
RF3	1	↓	0.990
RIBB	1	↓	0.556
RL10	1	↓	0.874
RL11	1	↓	0.950
RL7	1	↓	0.947
RL19	1	↓	0.916
RL1	1	↓	0.986
RL20	1	↓	0.917
RL27	1	↓	0.900
RL28	1	↓	0.756
RL29	1	↓	0.962
RL31	1	↓	0.887
RL32	1	↓	0.612
RL33	1	↓	0.802
RL9	1	↓	0.912
RS10	1	↓	0.809
RS11	1	↓	0.806
RS12	1	↓	0.896
RS13	1	↓	0.871
RS16	1	↓	0.794
RS18	1	↓	0.840
RS19	1	↓	0.862
RS20	1	↓	0.799
RS2	1	↓	0.890
RS3	1	↓	0.885
RS4	1	↓	0.895
RS5	1	↓	0.964
RS8	1	↓	0.961
RS9	1	↓	0.948
RIMM	1	↓	0.826
RNC	1	↓	0.903
RNH	1	↓	0.716
RPOA	1	↓	0.870
RPOZ	1	↓	0.911
RRF	1	↓	0.936
METK	1	↓	0.899
SELA	1	↓	0.789
GLYA	1	↓	0.967
DCTA	1	↓	0.563
TGT	1	↓	0.962
TIG	1	↓	0.823
TNAA	1	↓	0.445
TYSY	1	↓	0.772
UBIE	1	↓	0.780
YBEY	1	↓	0.887
YEBC	1	↓	0.938
YEEN	1	↓	0.799
PSRP	1	↓	0.916
RIMP	1	↓	0.968
YBAB	1	↓	0.887

YFBV	1	↓	0.665
YCFP	1	↓	0.832
URK	1	↓	0.874
UXAC	1	↓	0.606
NQOR	1	↓	0.963
SYS	1	↓	0.776
SYN	1	↓	0.877
SYT	1	↓	0.419
SYK1	1	↓	0.841
EPMA	1	↓	0.751
FRDC	1	↓	0.321
RPOC	1	↓	0.962
METJ	1	↓	0.680
RPOB	1	↓	0.921
FADR	1	↓	0.710
NANR	1	↓	0.857
YIHX	1	↓	0.914
SLYB	1	↓	0.947
MIPA	1	↓	0.769
PAL	1	↓	0.648
OMPW	1	↓	0.952
PA1	1	↓	0.799
TSX	1	↓	0.422
BAMA	1	↓	0.807
ATDA	1	↓	0.965
FABB	1	↓	0.974
CSPD	1	↓	0.307
CSPE	1	↓	0.576
DPO3B	1	↓	0.818
F16PA	1	↓	0.669
GLPC	1	↓	0.149
FTNA	1	↓	0.160
FTSZ	1	↓	0.930
E4PD	1	↓	0.931
GALM	1	↓	0.938
GLN1B	1	↓	0.724
GLPX	1	↓	0.795
MNTR	1	↓	0.754
CFA	1	↓	0.751
CITE	1	↓	0.764
RNG	1	↓	0.915
RBSK	1	↓	0.783
FKBB	1	↓	0.987
HPRT	1	↓	0.807
PTA	1	↓	0.881
PFLA	1	↓	0.574
DLDH	1	↓	0.981
DEAD	1	↓	0.498
ACCD	1	↓	0.972
FER	1	↓	0.610
FTSE	1	↓	0.964
FUCO	1	↓	0.412
GATD	1	↓	0.774
GLDA	1	↓	0.889

SERA	1	↓	0.976
LPTB	1	↓	0.972
ETTA	1	↓	0.869
CSPG	1	↓	0.726
CSPC	1	↓	0.616
GLNB	1	↓	0.932
RL13	1	↓	0.819
QMCA	1	↓	0.705
YAGU	1	↓	0.871
SDAC	1	↓	0.514
TDCC	1	↓	0.345
FABF	1	↓	0.843
HYPB	1	↓	0.100
YAJD	1	↓	0.000
YBAK	1	↓	0.968
YBDG	1	↓	0.977
IOJAP	1	↓	0.660
YBFE	1	↓	0.907
YCAR	1	↓	0.926
YCBJ	1	↓	0.916
PQIC	1	↓	0.987
YCCJ	1	↓	0.917
TUSE	1	↓	0.862
YCED	1	↓	0.884
LPOB	1	↓	0.929
PNTB	1	↓	0.939
ATPF	1	↓	0.759
ATPG	1	↓	0.891
ATPA	1	↓	0.865
ATPB	1	↓	0.892
ATMA	1	↓	0.849
BFR	1	↓	0.354
ACCA	1	↓	0.912
PCNB	1	↓	0.917
PGSA	1	↓	0.811
FTSA	1	↓	0.889
CLPA	1	↓	0.855
CORA	1	↓	1.000
DCUA	1	↓	0.163
DCUC	1	↓	0.359
COABC	1	↓	0.938
GARR	1	↓	0.606
DKSA	1	↓	0.825
DPS	1	↓	0.719
MENB	1	↓	0.827
YCHF	1	↓	0.980
ELBB	1	↓	0.853
FLIG	1	↓	0.194
BAMD	1	↓	0.936
FOCA	1	↓	0.598
FTSX	1	↓	0.833
FUMA	1	↓	0.481
ASPA	1	↓	0.363
FRDB	1	↓	0.098

ZUR	1	↓	0.857
GLRX4	1	↓	0.955
LGUL	1	↓	0.894
DNAB	1	↓	0.669
ERPA	1	↓	0.464
GLMU	1	↓	0.894
MBHL	1	↓	0.184
DBHA	1	↓	0.945
DBHB	1	↓	0.792
HNS	1	↓	0.822
STPA	1	↓	0.765
CRP	1	↓	0.776
GLPR	1	↓	0.774
PDHR	1	↓	0.877
CYTR	1	↓	0.580
CRA	1	↓	0.825
GNTR	1	↓	0.605
TDCA	1	↓	0.335
MPRA	1	↓	0.752
CECR	1	↓	0.938
YDCH	1	↓	0.998
EVGA	1	↓	0.716
YECF	1	↓	0.839
YFEC	1	↓	0.000
YFIA	1	↓	0.621
YAJG	1	↓	0.889
SUHB	1	↓	0.860
YIAF	1	↓	0.948
YIBL	1	↓	0.898
YIFE	1	↓	0.952
ZAPA	1	↓	0.998
YHCB	1	↓	0.952
PPID	1	↓	0.868
RL14	1	↓	0.825
RL23	1	↓	0.694
RS15	1	↓	0.781
YAJC	1	↓	0.973
ACRE	1	↓	0.959
AMPE	1	↓	0.636
APHA	1	↓	0.566
CHEY	1	↓	0.177
CITT	1	↓	0.749
CORC	1	↓	0.816
CPXA	1	↓	0.828
YNAI	1	↓	0.790
UVRY	1	↓	0.917
DGAL	1	↓	0.778
DSBC	1	↓	0.858
RSEP	1	↓	0.645
ELAA	1	↓	0.814
MIAB	1	↓	0.139
RIMO	1	↓	0.724
FABG	1	↓	0.974
FLGM	1	↓	0.254

FLIA	1	↓	0.064
FLIY	1	↓	0.786
GALU	1	↓	0.848
GYRB	1	↓	0.971
HYCA	1	↓	0.300
HYCI	1	↓	0.562
INGK	1	↓	0.826
MOTB	1	↓	0.000
APBC	1	↓	0.668
NAGC	1	↓	0.985
ZAPB	1	↓	0.711
GHOS	1	↓	0.470
TSAE	1	↓	0.984
NUOA	1	↓	0.650
NUOB	1	↓	0.399
NUOE	1	↓	0.366
NUOH	1	↓	0.563
NUOI	1	↓	0.467
NUPC	1	↓	0.834
NUPG	1	↓	0.567
YJDM	1	↓	0.658
POTB	1	↓	0.851
RNK	1	↓	0.443
RSEA	1	↓	0.976
SSEB	1	↓	0.987
WZZE	1	↓	0.695
YIBN	1	↓	0.880
RHO	1	↓	0.834
RIBF	1	↓	0.827
RL17	1	↓	0.870
RL21	1	↓	0.733
RL30	1	↓	0.984
RL6	1	↓	0.998
RS14	1	↓	0.847
RS17	1	↓	0.899
RS1	1	↓	0.779
SBCD	1	↓	0.908
SECB	1	↓	0.725
SECD	1	↓	0.825
SECE	1	↓	0.978
SECG	1	↓	0.898
SECY	1	↓	0.884
SODF	1	↓	0.195
CSDE	1	↓	0.986
TDCB	1	↓	0.206
TDCF	1	↓	0.361
RATA	1	↓	0.862
RL18	1	↓	0.933
IBPA	1	↓	0.665
SLMA	1	↓	0.935
SRKA	1	↓	0.894
PROP	1	↓	0.892
ISCX	1	↓	0.933
RLME	1	↓	0.919

GATY	1	↓	0.684
KBAZ	1	↓	0.530
EMTA	1	↓	0.866
EPTC	1	↓	0.833
EFTU1	1	↓	0.978
RLPA	1	↓	0.902
FTSY	1	↓	0.939
FADL	1	↓	0.972
SECA	1	↓	0.849
LPXB	1	↓	0.808
ARLY	1	↓	0.947
DAMX	1	↓	0.803
ARGO	1	↓	0.933
TDCD	1	↓	0.018
SYR	1	↓	0.956
MURF	1	↓	0.979
METH	1	↓	0.903
FDHE	1	↓	0.948
KATG	1	↓	0.502
RPOS	1	↓	0.890
PROV	1	↓	0.802
FUMB	1	↓	0.119
AMPP	1	↓	0.922
HELD	1	↓	0.954
FLIN	1	↓	0.065
PEPD	1	↓	0.951
PUR9	1	↓	0.759
MALQ	1	↓	0.767
HYCE	1	↓	0.103
MEND	1	↓	0.797
GUTQ	1	↓	0.830
GLMS	1	↓	0.518
MURG	1	↓	0.925
PNCB	1	↓	0.517
PARE	1	↓	0.872
PEPQ	1	↓	0.890
RNE	1	↓	0.944
LPXD	1	↓	0.684
SYC	1	↓	0.985
PCKA	1	↓	0.730
MUKB	1	↓	0.873
MUKE	1	↓	0.829
YCBB	1	↓	0.320
RIHC	1	↓	0.415
ISPA	1	↓	0.983
TRMA	1	↓	0.896
MALY	1	↓	0.850
KITH	1	↓	0.906
PPSA	1	↓	0.343
ECOT	1	↓	0.993
PSPE	1	↓	0.690
HEMH	1	↓	0.798
FMT	1	↓	0.805
ACCC	1	↓	0.854

HYPD	1	↓	0.191
HYPE	1	↓	0.320
YJIA	1	↓	0.540
UXUA	1	↓	0.583
CRL	1	↓	0.820
ACNA	1	↓	0.953
NTPPA	1	↓	1.000
YIDC	1	↓	0.847
ACUI	1	↓	0.875
YFED	1	↓	0.543
GCST	1	↓	0.478
TKT1	1	↓	0.763
EMRA	1	↓	0.808
STHA	1	↓	0.999
WECC	1	↓	0.610
RMLB2	1	↓	0.561
CYAY	1	↓	0.794
DCD	1	↓	0.964
ASMA	1	↓	0.654
METQ	1	↓	0.764
NRDD	1	↓	0.745
TREC	1	↓	0.440
MENC	1	↓	0.651
PEPT	1	↓	0.723
RNT	1	↓	0.859
SDHM	1	↓	0.079
METN	1	↓	0.910
GLMM	1	↓	0.800
ADEC	1	↓	0.451
YIEP	1	↓	0.959
LPTD	1	↓	0.866
YCIK	1	↓	0.897
NUOF	1	↓	0.645
CSQR	1	↓	0.629
FDOG	1	↓	0.199
GPH	1	↓	0.951
YJAG	1	↓	0.978
GCSP	1	↓	0.675
AIDB	1	↓	0.967
TORA	1	↓	0.345
TORC	1	↓	0.463
YEHZ	1	↓	0.990
BGLX	1	↓	0.869
NIKA	1	↓	0.769
NUOCD	1	↓	0.690
NUOG	1	↓	0.415
YEJF	1	↓	0.970
NAPA	1	↓	0.205
PTTBC	1	↓	0.395
ACNB	1	↓	0.598
RSMB	1	↓	0.831
PGM	1	↓	0.944
RLMN	1	↓	0.844
HYBD	1	↓	0.074

PTKB	1	↓	0.448
MODA	1	↓	0.911
YJJI	1	↓	0.707
DHAM	1	↓	0.942
UCPA	1	↓	0.447
PMRD	1	↓	0.354
PANZ	1	↓	0.975
YHII	1	↓	0.559
GADX	1	↓	0.683
KDGK	1	↓	0.827
YIAD	1	↓	0.941
RFAF	1	↓	0.911
RMLA1	1	↓	0.774
GLF	1	↓	0.956
WBBI	1	↓	0.830
WBBK	1	↓	0.817
RMLB1	1	↓	0.649
RMLD	1	↓	0.744
RLUB	1	↓	0.668
UXUB	1	↓	0.636
UXUR	1	↓	0.767
USPG	1	↓	0.663
PRMB	1	↓	0.895
YTFB	1	↓	0.848
QOR2	1	↓	0.834
YJHC	1	↓	0.728
IADA	1	↓	0.534
BTST	1	↓	0.493
YDFG	1	↓	0.887
OPGD	1	↓	0.811
PDXK	1	↓	0.959
RIHA	1	↓	0.560
TDCG	1	↓	0.182
TDCE	1	↓	0.222
OBG	1	↓	0.910
PQIB	1	↓	0.733
NANK	1	↓	0.788
PROQ	1	↓	0.841
CPOB	1	↓	0.981
HEXR	1	↓	0.704
YBHC	1	↓	0.854
YHGF	1	↓	0.902
FTSK	1	↓	0.710
MLC	1	↓	0.914
YAIL	1	↓	0.959
SFGH1	1	↓	0.743
IXTPA	1	↓	0.951
RATB	1	↓	0.754
RNLA	1	↓	0.550
NIFJ	1	↓	0.704
DLHH	1	↓	0.930
PTHC	1	↓	0.774
PTHB	1	↓	0.828
TRUB	1	↓	0.668

RL2	1	↓	0.854
RL3	1	↓	0.808
KGUA	1	↓	0.925
RL24	1	↓	0.818
LIPA	1	↓	0.542
RL4	1	↓	0.942
RL22	1	↓	0.904
RMLA2	1	↓	0.827
RL5	1	↓	0.872
ISPE	1	↓	0.940
ISPG	1	↓	0.797
ISPH	1	↓	0.848
KUP	1	↓	0.650
GADE	1	↓	0.636
MLAF	1	↓	0.944
YHBS	1	↓	0.819
YDCY	1	↓	0.750
CNU	1	↓	0.915
YOAC	1	↓	0.655
YFCL	1	↓	0.719
YGFM	1	↓	0.000
YRAP	1	↓	0.990
MLAB	1	↓	0.798
YHEO	1	↓	0.859
YQCA	1	↓	0.903
YFCD	1	↓	0.880
YGEY	1	↓	0.090
DIAA	1	↓	0.919
RSMI	1	↓	0.945
YHAJ	1	↓	0.955
CUTC	1	↓	0.834
SRA	1	↓	0.974
RS21	1	↓	0.663
ATPL	1	↓	0.800
NFI	1	↓	0.931
RL25	1	↓	0.912
MDTJ	1	↓	0.759
IF1	1	↓	0.737
CITD	1	↓	0.844
RCSF	1	↓	0.967
TATB	1	↓	0.929
TATA	1	↓	0.734
KAD	1	↓	0.780
APT	1	↓	0.953
MBHS	1	↓	0.077
MBHT	1	↓	0.000
LPP	1	↓	0.702
PTNAB	1	↓	0.980
PTNC	1	↓	0.938
PTKA	1	↓	0.374
PTSN	1	↓	0.915
PTKC	1	↓	0.479
DMSD	1	↓	0.450
FCTA	1	↓	0.933

YAGE	1	↓	0.754
CILA	1	↓	0.643
PXPC	1	↓	0.811
YBHG	1	↓	0.885
RLMF	1	↓	0.980
LTAE	1	↓	0.943
HCR	1	↓	0.285
HCP	1	↓	0.238
YBJX	1	↓	0.417
RLMI	1	↓	0.977
YCDX	1	↓	0.985
YCDY	1	↓	0.958
JAYE	1	↓	0.962
DHAL	1	↓	0.851
OPPD	1	↓	0.937
RACR	1	↓	0.881
TAM	1	↓	0.978
YDGH	1	↓	0.962
TSAB	1	↓	0.915
MTFA	1	↓	0.514
WZZB	1	↓	0.945
LPXT	1	↓	0.908
MLAA	1	↓	0.841
YFDI	1	↓	0.727
YFEX	1	↓	0.607
MAO2	1	↓	0.771
PDXY	1	↓	0.789
ZIPA	1	↓	1.000
GTRB	1	↓	0.730
DTPA	1	↓	0.972
YDEN	1	↓	0.608
HLYE	1	↓	0.998
MSCK	1	↓	0.959
MPPA	1	↓	0.775
YNFE	1	↓	0.303
DXS	1	↓	0.627
YDJN	1	↓	0.743
YFCF	1	↓	0.879
YDHQ	1	↓	0.882
ANMK	1	↓	0.991
YDJF	1	↓	0.960
OPPF	1	↓	0.660
BAMB	1	↓	0.921
YNFF	1	↓	0.018
MAA	1	↓	0.350
YDGA	1	↓	0.908
YNJE	1	↓	0.271
YGEV	1	↓	0.855
YGEW	1	↓	0.079
YGFK	1	↓	0.274
YAJL	1	↓	0.859
DINJ	1	↓	0.787

TableS4: Proteomic data higher abundance in LB/TMAO against LB/TMAO + 150 µM DIP

The detectable proteins that identified by proteomics analysis with and without 150 mM 2,2-DIP. The listed protein names with corresponding fold changes in the presence of 150 mM 2,2-DIP compared to without 150 mM 2,2-DIP. The strain was cultivated for 4 h in LB media at 37 °C with 20 mM TMAO. Green arrow shows increased fold.

**Protein name**	**LB+TMAO**	**LB+TMAO+150 DIP (Fold changes)**
6PGD	1	↑ 1.258
DHE4	1	↑ 1.526
DHNA	1	↑ 4.573
SODM	1	↑ 3.658
RIR1	1	↑ 2.879
AAT	1	↑ 1.214
PTM3C	1	↑ 1.567
AK2H	1	↑ 1.194
RPOD	1	↑ 1.209
DPO1	1	↑ 1.090
LEP	1	↑ 1.491
AMPC	1	↑ 1.074
CAPP	1	↑ 1.058
SDHD	1	↑ 3.696
MANA	1	↑ 1.044
SYW	1	↑ 1.138
SYI	1	↑ 1.126
SYA	1	↑ 1.090
SYM	1	↑ 1.065
SYGA	1	↑ 1.039
SYGB	1	↑ 1.095
SYQ	1	↑ 1.114
ASNA	1	↑ 1.034
RS7	1	↑ 1.062
RL15	1	↑ 1.070
MALF	1	↑ 1.029
PBPA	1	↑ 1.159
PBPB	1	↑ 1.241
RBSB	1	↑ 1.239
UVRD	1	↑ 1.050
MIOC	1	↑ 1.228
DAPB	1	↑ 1.118
GUAA	1	↑ 1.587
FRSA	1	↑ 1.590
GSHB	1	↑ 1.189
TYRB	1	↑ 1.419
SYE	1	↑ 1.303
AMPN	1	↑ 1.132
KDSB	1	↑ 1.189
RBSD	1	↑ 1.475
RBSA	1	↑ 1.227
PYRC	1	↑ 1.075
AROD	1	↑ 1.034
PDXB	1	↑ 1.165
APAH	1	↑ 1.266
ILVD	1	↑ 1.116
ILVC	1	↑ 1.152
FEPA	1	↑ 46.995
DLD	1	↑ 1.179
BTUE	1	↑ 1.173
TOP1	1	↑ 1.022
ERA	1	↑ 1.084

BIRA	1	↑	1.048
DPO3X	1	↑	1.288
GSHR	1	↑	1.464
METC	1	↑	1.665
ODP2	1	↑	1.336
CCA	1	↑	1.154
DICA	1	↑	1.179
DUT	1	↑	1.204
FHUA	1	↑	49.436
HIS8	1	↑	1.209
HIS7	1	↑	1.058
HIS2	1	↑	1.023
RSMA	1	↑	1.016
OMPC	1	↑	1.000
PFKB	1	↑	2.051
POXB	1	↑	2.150
RF2	1	↑	1.133
PPA	1	↑	2.154
SYFB	1	↑	1.030
TYRR	1	↑	1.061
AROB	1	↑	1.011
TRUA	1	↑	1.090
TYPH	1	↑	1.175
SYL	1	↑	1.043
DDLB	1	↑	1.165
TDH	1	↑	1.078
FOLC	1	↑	1.279
NIRB	1	↑	2.919
PYRF	1	↑	1.292
SYFA	1	↑	1.050
MURB	1	↑	1.043
USG	1	↑	1.371
DNAJ	1	↑	1.063
AK3	1	↑	1.926
PTFX1	1	↑	1.852
EX3	1	↑	1.013
AVTA	1	↑	1.327
RND	1	↑	1.094
ASNB	1	↑	1.281
PFLB	1	↑	2.286
MTLD	1	↑	1.810
ARGT	1	↑	1.221
UVRA	1	↑	1.324
ACKA	1	↑	1.001
ACP	1	↑	1.058
END4	1	↑	1.477
AROA	1	↑	1.412
ASSY	1	↑	1.059
CH60	1	↑	1.287
CH10	1	↑	1.347
PNCC	1	↑	1.195
CLPP	1	↑	1.159
HSLU	1	↑	1.122
COAA	1	↑	1.026
COAD	1	↑	1.183
CYSZ	1	↑	1.458
DEOB	1	↑	4.335

DEOC	1	↑	1.452
DAPA	1	↑	1.100
HSCB	1	↑	2.650
DSBB	1	↑	1.192
DTD	1	↑	1.018
EFPL	1	↑	1.118
EFTS	1	↑	1.070
DER	1	↑	1.242
ENGB	1	↑	1.108
ENO	1	↑	1.126
FABZ	1	↑	1.026
FABH	1	↑	1.190
G6PI	1	↑	1.601
GCH1	1	↑	2.132
GLK	1	↑	1.061
MRAY	1	↑	1.222
GREA	1	↑	1.009
GSH1	1	↑	1.068
IHFA	1	↑	1.127
IHFB	1	↑	1.265
HSLO	1	↑	1.202
DNAK	1	↑	1.619
HSCA	1	↑	1.201
HTPG	1	↑	1.418
NIKR	1	↑	1.371
KDSA	1	↑	1.114
KPRS	1	↑	1.037
LPXC	1	↑	1.108
NTPPB	1	↑	1.296
MINE	1	↑	1.020
MSCL	1	↑	1.525
MSRA	1	↑	1.101
NAGB	1	↑	1.283
NUSB	1	↑	1.048
ORN	1	↑	1.126
PAND	1	↑	1.093
PDXJ	1	↑	1.188
PFKA	1	↑	1.840
PGK	1	↑	1.541
PIMT	1	↑	1.024
PLSB	1	↑	1.150
IPYR	1	↑	1.191
PPK1	1	↑	1.494
NADK	1	↑	1.136
PROB	1	↑	1.021
PURA	1	↑	1.052
PYRG	1	↑	1.017
PYRH	1	↑	1.338
QUEA	1	↑	1.070
RBFA	1	↑	1.064
RECA	1	↑	1.032
RECR	1	↑	1.588
RIBA	1	↑	1.184
RIBB	1	↑	1.213
RL10	1	↑	1.032
RL11	1	↑	1.012
RL7	1	↑	1.021

RL19	1	↑	1.039
RL1	1	↑	1.041
RL20	1	↑	1.000
RL28	1	↑	1.016
RL29	1	↑	1.164
RL31	1	↑	1.000
RL34	1	↑	1.075
RS10	1	↑	1.038
RS13	1	↑	1.048
RS16	1	↑	1.030
RS19	1	↑	1.028
RS2	1	↑	1.033
RS5	1	↑	1.060
RS8	1	↑	1.008
RS9	1	↑	1.070
RNC	1	↑	1.097
RNH	1	↑	1.157
RPIA	1	↑	1.294
RPOA	1	↑	1.020
RRF	1	↑	1.038
METK	1	↑	1.752
SELA	1	↑	1.092
SSRP	1	↑	1.062
TOLB	1	↑	1.193
TPIS	1	↑	1.223
TPX	1	↑	1.003
TALA	1	↑	2.094
TALB	1	↑	1.404
TRMD	1	↑	1.207
TUSA	1	↑	1.341
RAPZ	1	↑	1.153
YBEY	1	↑	1.005
PSRP	1	↑	1.085
YBAB	1	↑	1.022
YBJQ	1	↑	1.059
YGFB	1	↑	1.385
YMDB	1	↑	1.820
YACL	1	↑	2.004
YAJQ	1	↑	1.507
URK	1	↑	1.027
UVRB	1	↑	1.605
EX7S	1	↑	1.124
YIHI	1	↑	1.447
YAAA	1	↑	1.318
RLMH	1	↑	1.086
YBED	1	↑	1.249
RHLB	1	↑	1.042
PSD	1	↑	1.098
YCIU	1	↑	1.634
YEEX	1	↑	1.472
SYK1	1	↑	1.148
SYK2	1	↑	1.605
FETP	1	↑	1.179
RRAA	1	↑	1.138
YCJF	1	↑	1.403
PRMA	1	↑	1.300
RPOC	1	↑	1.021

RL19	1	↑	1.039
RL1	1	↑	1.041
RL20	1	↑	1.000
RL28	1	↑	1.016
RL29	1	↑	1.164
RL31	1	↑	1.000
RL34	1	↑	1.075
RS10	1	↑	1.038
RS13	1	↑	1.048
RS16	1	↑	1.030
RS19	1	↑	1.028
RS2	1	↑	1.033
RS5	1	↑	1.060
RS8	1	↑	1.008
RS9	1	↑	1.070
RNC	1	↑	1.097
RNH	1	↑	1.157
RPIA	1	↑	1.294
RPOA	1	↑	1.020
RRF	1	↑	1.038
METK	1	↑	1.752
SELA	1	↑	1.092
SSRP	1	↑	1.062
TOLB	1	↑	1.193
TPIS	1	↑	1.223
TPX	1	↑	1.003
TALA	1	↑	2.094
TALB	1	↑	1.404
TRMD	1	↑	1.207
TUSA	1	↑	1.341
RAPZ	1	↑	1.153
YBEY	1	↑	1.005
PSRP	1	↑	1.085
YBAB	1	↑	1.022
YBJQ	1	↑	1.059
YGFB	1	↑	1.385
YMDB	1	↑	1.820
YACL	1	↑	2.004
YAJQ	1	↑	1.507
URK	1	↑	1.027
UVRB	1	↑	1.605
EX7S	1	↑	1.124
YIHI	1	↑	1.447
YAAA	1	↑	1.318
RLMH	1	↑	1.086
YBED	1	↑	1.249
RHLB	1	↑	1.042
PSD	1	↑	1.098
YCIU	1	↑	1.634
YEEX	1	↑	1.472
SYK1	1	↑	1.148
SYK2	1	↑	1.605
FETP	1	↑	1.179
RRAA	1	↑	1.138
YCJF	1	↑	1.403
PRMA	1	↑	1.300
RPOC	1	↑	1.021

RBN	1	↑	1.157
RPOB	1	↑	1.029
NANR	1	↑	1.059
YQGE	1	↑	1.341
YFBU	1	↑	1.450
YJGA	1	↑	1.383
YIDA	1	↑	1.449
YBGC	1	↑	1.088
BLC	1	↑	1.206
SLYB	1	↑	1.086
OMPA	1	↑	1.159
OMPX	1	↑	1.905
BAME	1	↑	1.028
RIMJ	1	↑	1.124
ALKH	1	↑	1.349
ALAA	1	↑	1.262
ASPG1	1	↑	1.187
ALF1	1	↑	1.564
FUR	1	↑	1.081
G3P1	1	↑	1.958
E4PD	1	↑	1.125
GLPA	1	↑	1.574
GSTA	1	↑	1.030
CYSE	1	↑	1.094
DAPD	1	↑	1.283
MNTR	1	↑	2.764
CYSB	1	↑	1.042
LDCI	1	↑	3.515
CFA	1	↑	1.502
CITE	1	↑	10.187
GCVR	1	↑	1.310
NIRD	1	↑	2.248
RNG	1	↑	1.021
CMPDT	1	↑	1.091
PHOL	1	↑	1.031
PHOU	1	↑	1.172
SLYD	1	↑	1.217
FKBB	1	↑	1.013
PPIC	1	↑	1.280
LON	1	↑	1.258
HPRT	1	↑	1.092
XGPT	1	↑	1.113
PTA	1	↑	1.085
DLDH	1	↑	1.096
TRXB	1	↑	1.172
ACCD	1	↑	1.081
ADHE	1	↑	3.497
DHAS	1	↑	1.301
FTSE	1	↑	1.120
GATD	1	↑	1.004
SERA	1	↑	1.026
TAS	1	↑	1.271
YBIT	1	↑	1.174
LPTB	1	↑	1.055
ULAR	1	↑	1.002
IBAG	1	↑	1.126
MREB	1	↑	1.331

CSPG	1	↑	1.175
GLNB	1	↑	1.067
PTHP	1	↑	1.468
RL13	1	↑	1.012
THIO	1	↑	1.029
RLUC	1	↑	1.101
RSUA	1	↑	1.230
YOBA	1	↑	1.337
YEAY	1	↑	1.302
YAEQ	1	↑	1.254
YAGU	1	↑	1.391
GALF	1	↑	1.239
USPD	1	↑	1.736
USPE	1	↑	1.309
ARTP	1	↑	1.106
SAPF	1	↑	2.159
FTSH	1	↑	1.125
FRMR	1	↑	1.022
YBAK	1	↑	1.255
YBCJ	1	↑	1.364
YBDF	1	↑	1.074
YBEL	1	↑	1.280
YBFE	1	↑	1.133
YBIS	1	↑	1.390
YCAR	1	↑	1.104
PQIC	1	↑	1.110
YCCF	1	↑	1.046
TUSE	1	↑	1.088
EFEO	1	↑	1.525
YCEB	1	↑	1.426
YCED	1	↑	1.007
YCGL	1	↑	1.121
YMGD	1	↑	1.627
YCII	1	↑	1.714
LAPB	1	↑	1.341
YCIN	1	↑	1.536
ACYP	1	↑	1.043
ALF	1	↑	1.872
ILVE	1	↑	1.233
PUR8	1	↑	1.095
AROG	1	↑	1.407
ATMA	1	↑	1.264
HFLK	1	↑	1.041
BCCP	1	↑	1.049
CDD	1	↑	3.035
PGSA	1	↑	1.015
CORA	1	↑	1.087
CYOB	1	↑	1.933
CYOA	1	↑	1.818
CYDA	1	↑	1.148
CYDB	1	↑	1.179
CYSK	1	↑	1.388
DEOD	1	↑	2.251
COABC	1	↑	1.200
DYR	1	↑	1.193
DPS	1	↑	1.374
EXBB	1	↑	34.771

TOLQ	1	↑	1.219
EXBD	1	↑	4.703
TOLR	1	↑	1.085
FLAW	1	↑	1.236
KDSC	1	↑	1.004
SURA	1	↑	1.075
DHPS	1	↑	1.289
FOCA	1	↑	1.410
FTSX	1	↑	1.266
G6PD	1	↑	1.338
GLRX2	1	↑	1.438
GLRX3	1	↑	1.209
GLRX4	1	↑	1.235
KDTA	1	↑	1.013
LGUL	1	↑	1.017
YIBF	1	↑	1.231
SSPA	1	↑	1.180
GSTB	1	↑	1.383
HEM2	1	↑	1.562
ROB	1	↑	1.336
LRP	1	↑	1.178
PDHR	1	↑	1.040
ALLR	1	↑	1.127
GNTR	1	↑	1.053
PURR	1	↑	1.188
YJDC	1	↑	1.331
LPXL	1	↑	1.030
YDHR	1	↑	1.400
YEAG	1	↑	1.453
CUSR	1	↑	1.429
YECA	1	↑	1.234
YECJ	1	↑	1.469
YEEZ	1	↑	1.303
YFCZ	1	↑	1.861
ISPB	1	↑	1.122
IVY	1	↑	1.897
KPYK1	1	↑	1.775
TESA	1	↑	1.191
OSMB	1	↑	2.522
OSME	1	↑	1.331
LPTE	1	↑	1.248
LOLC	1	↑	1.084
KBP	1	↑	2.019
YGFZ	1	↑	1.123
EDD	1	↑	1.122
SUHB	1	↑	1.252
IMDH	1	↑	1.967
YECD	1	↑	1.183
YIAF	1	↑	1.095
YIBL	1	↑	1.141
YIFE	1	↑	1.109
YIHD	1	↑	1.027
YGAM	1	↑	1.365
ZAPA	1	↑	1.320
YGGE	1	↑	1.445
YGGN	1	↑	1.174
YGIB	1	↑	1.006

YGIM	1	↑	1.244
YGIN	1	↑	1.702
YGIW	1	↑	2.479
YHBW	1	↑	1.814
MLAC	1	↑	1.099
LPTC	1	↑	1.398
YHCB	1	↑	1.118
YHFA	1	↑	1.695
YHHA	1	↑	2.685
RL14	1	↑	1.093
RS15	1	↑	1.064
TRMJ	1	↑	1.419
AHPC	1	↑	1.135
AMPE	1	↑	1.068
MAP1	1	↑	2.101
YPDB	1	↑	2.415
BCP	1	↑	1.162
CPXR	1	↑	1.038
CYSG	1	↑	1.477
DACA	1	↑	1.122
YOAB	1	↑	1.094
ARCB	1	↑	1.167
DCM	1	↑	1.169
DCRB	1	↑	1.306
DEGS	1	↑	1.391
DSBC	1	↑	1.122
RSEP	1	↑	1.051
ELAB	1	↑	1.897
EUTB	1	↑	1.112
FABI	1	↑	1.262
GLCG	1	↑	1.094
GSP	1	↑	2.242
GYRA	1	↑	1.089
GYRB	1	↑	1.071
HDEA	1	↑	1.630
HDEB	1	↑	2.127
HDED	1	↑	3.619
HDHA	1	↑	1.071
SKP	1	↑	1.300
RSSB	1	↑	1.201
CPDA	1	↑	1.151
K1PF	1	↑	5.253
MDAB	1	↑	1.534
MTNN	1	↑	1.253
NAGA	1	↑	1.806
NAGC	1	↑	1.103
NAGD	1	↑	1.160
ZAPB	1	↑	1.116
YJBQ	1	↑	1.521
YJBR	1	↑	1.785
TSAE	1	↑	1.365
RRAB	1	↑	1.405
NUON	1	↑	2.502
NUPC	1	↑	1.107
NUSG	1	↑	1.105
ODP1	1	↑	1.270
OSMY	1	↑	1.446

PARC	1	↑	1.008
PDXH	1	↑	1.296
PMBA	1	↑	1.291
POTB	1	↑	1.227
PPIA	1	↑	1.010
PPX	1	↑	1.008
PSIF	1	↑	2.295
YBBO	1	↑	1.022
YCFH	1	↑	1.317
YCIO	1	↑	1.268
RISA	1	↑	1.248
RMF	1	↑	2.408
ROF	1	↑	1.523
HPF	1	↑	1.417
RSEA	1	↑	1.161
RSEB	1	↑	1.295
SEQA	1	↑	1.093
SSEB	1	↑	1.774
SOHB	1	↑	1.339
RELA	1	↑	1.127
RIBF	1	↑	1.094
RL6	1	↑	1.030
RS17	1	↑	1.152
PSTS	1	↑	1.110
YGHA	1	↑	1.469
SECG	1	↑	1.108
SECY	1	↑	1.075
SERB	1	↑	1.151
SLT	1	↑	1.050
SODC	1	↑	2.000
CHRR	1	↑	1.677
CSDE	1	↑	1.786
THIL	1	↑	1.028
TESB	1	↑	1.016
THIO2	1	↑	1.082
TLDD	1	↑	1.133
TRMH	1	↑	1.135
YFIF	1	↑	1.226
TRML	1	↑	1.334
SYY	1	↑	1.099
YHBY	1	↑	1.119
RATA	1	↑	1.043
PPNP	1	↑	1.132
OSMC	1	↑	2.087
PROP	1	↑	1.182
ISCX	1	↑	1.517
MSCS	1	↑	2.511
DEGP	1	↑	1.422
EPTC	1	↑	1.137
RLPA	1	↑	1.308
HIS4	1	↑	1.064
NARU	1	↑	2.903
ARLY	1	↑	1.414
SYR	1	↑	1.098
AROC	1	↑	1.345
UNG	1	↑	1.043
UDP	1	↑	2.352

YBHB	1	↑	1.816
GLPB	1	↑	1.605
GLPD	1	↑	4.955
FECA	1	↑	4306.730
RIML	1	↑	1.208
PROV	1	↑	1.020
MURD	1	↑	1.158
HELD	1	↑	1.345
DNLJ	1	↑	1.155
DGTP	1	↑	1.232
AROE	1	↑	1.207
MIAA	1	↑	1.167
SELD	1	↑	1.184
SYP	1	↑	1.246
YJDN	1	↑	1.088
CYSP	1	↑	1.069
MREC	1	↑	1.122
MEND	1	↑	1.252
NFSA	1	↑	1.665
MURG	1	↑	1.028
BETB	1	↑	2.574
MURC	1	↑	1.073
MINC	1	↑	1.164
NADE	1	↑	1.530
PDXA	1	↑	1.039
PARE	1	↑	1.023
FIC	1	↑	2.029
PTFBC	1	↑	3.938
SPEA	1	↑	1.052
RNI	1	↑	1.321
YCIF	1	↑	1.132
YCAC	1	↑	1.729
RNR	1	↑	1.208
SRMB	1	↑	1.063
LPXD	1	↑	1.078
YBHA	1	↑	1.063
SYD	1	↑	1.184
ASNB	1	↑	1.431
MURE	1	↑	1.084
CYSQ	1	↑	1.161
GABT	1	↑	1.180
ADD	1	↑	1.336
MURI	1	↑	1.200
MALY	1	↑	1.006
SERC	1	↑	1.365
ECOT	1	↑	1.000
YICC	1	↑	1.226
PPIB	1	↑	1.185
FMT	1	↑	1.007
GSA	1	↑	1.126
ARGE	1	↑	1.191
MUTS	1	↑	1.146
DCP	1	↑	1.271
ACCC	1	↑	1.019
FOLD	1	↑	1.357
HMP	1	↑	1.351
ASCG	1	↑	1.069

RP54	1	↑	1.248
TEHB	1	↑	1.537
MNME	1	↑	1.462
GABD	1	↑	1.287
UBIH	1	↑	1.039
UBII	1	↑	1.139
NTPPA	1	↑	1.218
RIBD	1	↑	1.264
GPPA	1	↑	1.000
METE	1	↑	161.776
MSYB	1	↑	1.639
RFAP	1	↑	1.127
MNMA	1	↑	1.046
HFLD	1	↑	1.159
RHLE	1	↑	1.160
LOIP	1	↑	1.045
APPC	1	↑	1.129
AMY2	1	↑	1.140
MAO1	1	↑	1.234
RFAY	1	↑	1.017
GLND	1	↑	1.752
AHR	1	↑	2.237
OPDA	1	↑	1.184
ROXA	1	↑	1.169
ACSA	1	↑	1.524
CYAY	1	↑	1.016
YIGL	1	↑	1.175
QOR1	1	↑	1.430
HOLA	1	↑	1.481
METQ	1	↑	1.163
FENR	1	↑	1.291
ALR2	1	↑	1.177
YCGB	1	↑	1.352
CYDD	1	↑	1.177
FTSN	1	↑	1.055
YCEH	1	↑	1.068
DCUP	1	↑	1.263
NADC	1	↑	1.106
RNT	1	↑	1.260
GREB	1	↑	1.353
YBIB	1	↑	1.056
METN	1	↑	1.148
RNB	1	↑	1.215
ARTI	1	↑	1.137
ARTJ	1	↑	1.115
DKGB	1	↑	2.025
YAFC	1	↑	1.095
GLNE	1	↑	1.220
3PASE	1	↑	1.094
MFD	1	↑	1.487
PANB	1	↑	1.571
YEDD	1	↑	1.004
KCH	1	↑	1.028
THTM	1	↑	1.085
ACRF	1	↑	1.093
YICH	1	↑	1.198
HCHA	1	↑	1.899

PRPC	1	↑	1.087
PANC	1	↑	1.545
OTSA	1	↑	1.347
OTSB	1	↑	1.734
DJLA	1	↑	1.447
NNR	1	↑	1.043
YCIK	1	↑	1.160
LPLA	1	↑	1.132
TYPA	1	↑	1.317
YIIM	1	↑	1.413
YIIS	1	↑	2.053
GPH	1	↑	1.001
NUDC	1	↑	1.105
YJAG	1	↑	1.218
DUSA	1	↑	1.103
YEIR	1	↑	1.091
OPGG	1	↑	1.024
YEBE	1	↑	1.464
YEBF	1	↑	1.458
AIDB	1	↑	2.125
YEHS	1	↑	1.001
YOHF	1	↑	1.757
TKT2	1	↑	1.947
RLUD	1	↑	1.443
POLOX	1	↑	1.196
FEOB	1	↑	166.918
NDPA	1	↑	1.252
YDCF	1	↑	1.112
AHPF	1	↑	1.352
CUEO	1	↑	1.866
CBPA	1	↑	2.118
ZAPD	1	↑	1.056
RDGC	1	↑	1.493
YADG	1	↑	1.077
YEGD	1	↑	1.362
RLMN	1	↑	1.016
CLCA	1	↑	1.146
PURU	1	↑	1.610
PEPB	1	↑	1.578
NUDK	1	↑	1.895
RIR4	1	↑	14.315
PT1P	1	↑	1.190
SLP	1	↑	2.184
DCTR	1	↑	1.332
MODA	1	↑	1.102
DHAM	1	↑	1.406
PANZ	1	↑	1.025
ZNTA	1	↑	1.328
RLMJ	1	↑	1.037
MDTE	1	↑	1.881
MDTF	1	↑	1.955
YHJD	1	↑	1.125
YHJG	1	↑	1.330
YHJJ	1	↑	1.291
EPTB	1	↑	1.088
YIAD	1	↑	1.273
GHRB	1	↑	1.253

YIAJ	1	↑	1.254
GPMI	1	↑	1.500
GLF	1	↑	1.025
MPL	1	↑	1.083
CYSJ	1	↑	2.500
NFSB	1	↑	1.322
YEAD	1	↑	2.489
ERFK	1	↑	1.475
PRMB	1	↑	1.010
ALSB	1	↑	1.197
YJGR	1	↑	2.028
YJHU	1	↑	1.156
RSMC	1	↑	1.077
YJJU	1	↑	1.428
NCPP	1	↑	1.494
ADHP	1	↑	3.341
YDFG	1	↑	1.162
GNTT	1	↑	1.826
RCSD	1	↑	1.282
NLPE	1	↑	1.205
MTOX	1	↑	1.043
MLTB	1	↑	1.223
AZOR	1	↑	1.192
YQJG	1	↑	1.435
OBG	1	↑	1.097
PQIB	1	↑	1.019
UUP	1	↑	1.003
KDSD	1	↑	1.222
LPOA	1	↑	1.042
YHBO	1	↑	2.228
FKBA	1	↑	1.084
DXR	1	↑	1.121
LUXS	1	↑	1.912
TSAC	1	↑	1.417
NUDE	1	↑	1.143
CPOB	1	↑	1.054
YEDI	1	↑	1.130
YBHC	1	↑	1.069
YHHW	1	↑	1.676
YHHX	1	↑	1.283
FTSK	1	↑	1.064
YECM	1	↑	1.216
YGGU	1	↑	1.578
DCLZ	1	↑	1.496
LDHD	1	↑	1.414
6PGL	1	↑	1.860
CSIE	1	↑	1.221
YGAP	1	↑	1.607
DLHH	1	↑	1.126
RAPA	1	↑	1.224
RL2	1	↑	1.029
KGUA	1	↑	1.382
HIS5	1	↑	1.101
SPEB	1	↑	1.282
LIPB	1	↑	1.641
MSBA	1	↑	1.075
HIS1	1	↑	1.101

SYH	1	↑	1.044
RL22	1	↑	1.038
LOLA	1	↑	1.364
LOLB	1	↑	1.465
CAN	1	↑	2.358
RISB	1	↑	1.148
RMLA2	1	↑	1.035
FLAV	1	↑	1.207
RL5	1	↑	1.063
OPGH	1	↑	1.081
ISPE	1	↑	1.235
ISPG	1	↑	1.078
GPMA	1	↑	6.638
YAEH	1	↑	1.093
NFUA	1	↑	2.421
GMHA	1	↑	1.133
GMHBB	1	↑	1.205
GADC	1	↑	2.293
CBPM	1	↑	1.876
CLPB	1	↑	2.608
YPFJ	1	↑	1.314
YDCL	1	↑	1.329
YDCY	1	↑	1.259
YDFZ	1	↑	1.701
YDIH	1	↑	1.545
YDIZ	1	↑	1.577
YEAK	1	↑	1.102
YEBV	1	↑	2.049
YODD	1	↑	2.625
RCNB	1	↑	1.598
YQJD	1	↑	1.273
YQJE	1	↑	1.394
MLAD	1	↑	1.058
YHEO	1	↑	1.288
YGDI	1	↑	2.097
YGDR	1	↑	1.096
YQCA	1	↑	1.028
BEPA	1	↑	1.096
PLPHP	1	↑	1.081
YFCE	1	↑	1.455
CUTC	1	↑	1.059
HLDD	1	↑	1.377
GRCA	1	↑	6.124
MALK	1	↑	1.239
SRA	1	↑	1.256
YJBJ	1	↑	2.040
GLRX1	1	↑	1.200
NFI	1	↑	1.485
RL25	1	↑	1.002
MDTJ	1	↑	1.216
BAER	1	↑	1.210
CITD	1	↑	5.808
RCSF	1	↑	1.294
CUTA	1	↑	1.187
APT	1	↑	1.186
YTFE	1	↑	5.390
LPP	1	↑	1.092

PTGA	1	↑	1.272
PTNC	1	↑	1.186
PTND	1	↑	1.019
PTFAH	1	↑	4.374
DCEB	1	↑	2.156
RIR2	1	↑	2.372
YAHK	1	↑	1.760
YAHO	1	↑	1.283
CILA	1	↑	18.999
UBIF	1	↑	1.394
YBHG	1	↑	1.228
YBJI	1	↑	1.081
YBJP	1	↑	1.509
YCAO	1	↑	1.647
GLO22	1	↑	1.282
RLMKL	1	↑	1.092
YCCU	1	↑	1.474
RLMI	1	↑	1.328
GHRA	1	↑	1.167
LOLD	1	↑	1.134
LOLE	1	↑	1.501
PLIG	1	↑	1.331
LDCA	1	↑	1.216
YCJX	1	↑	1.022
YDCJ	1	↑	1.004
CURA	1	↑	1.394
YNCE	1	↑	1882.822
LSRB	1	↑	1.034
LSRF	1	↑	1.177
TAM	1	↑	1.485
YDGH	1	↑	1.053
YDHF	1	↑	1.259
YNHG	1	↑	2.316
YEAH	1	↑	1.461
YEAO	1	↑	1.244
TSAB	1	↑	1.322
MSRC	1	↑	1.197
CMOA	1	↑	1.284
CMOB	1	↑	1.202
DCYD	1	↑	1.039
MPGP	1	↑	1.756
YEGH	1	↑	1.118
YEGP	1	↑	1.927
YEGQ	1	↑	1.435
LPXT	1	↑	1.026
YFEY	1	↑	1.123
YFFS	1	↑	1.142
YFGD	1	↑	1.168
HLDE	1	↑	1.059
YDHS	1	↑	1.425
PDXY	1	↑	1.061
DSBG	1	↑	1.161
RCLA	1	↑	1.247
GCS2	1	↑	1.204
PRPD	1	↑	1.628
NEMA	1	↑	1.073
DTPA	1	↑	1.086

MSCK	1	↑	1.266
YDGJ	1	↑	1.091
CNOX	1	↑	1.459
GLSA1	1	↑	2.222
YDJN	1	↑	1.258
PRPB	1	↑	2.056
YDHQ	1	↑	1.013
YAHJ	1	↑	1.161
ASTC	1	↑	1.925
HXPA	1	↑	1.159
GNSB	1	↑	1.119
YBAY	1	↑	1.336
THII	1	↑	1.137
YAJO	1	↑	1.408
SPY	1	↑	1.791
QUEC	1	↑	1.333
BAMB	1	↑	1.011
YDGA	1	↑	1.076
CINAL	1	↑	1.257
FSAA	1	↑	1.271
YIBT	1	↑	1.762
BGLA	1	↑	1.091
YGHU	1	↑	1.408
YQHD	1	↑	1.490
DKGA	1	↑	2.243
UBIK	1	↑	1.442
YQJH	1	↑	24.944
ISPD	1	↑	1.167
QUEF	1	↑	1.142
CSDA	1	↑	1.641
YAJL	1	↑	1.329
DINJ	1	↑	1.008
YAFV	1	↑	1.192
YQJK	1	↑	1.336
TRUD	1	↑	1.170
COPA	1	↑	3.338

Table S5: Proteomic data lower abundance in LB/TMAO against LB/TMAO + 150 µM DIP

The detectable proteins that identified by proteomics analysis with and without 150 mM 2,2-DIP. The listed protein names with corresponding fold changes in the presence of 150 mM 2,2-DIP compared to without 150 mM 2,2-DIP. The strain was cultivated for 4 h in LB media at 37 °C with 20 mM TMAO. Red arrow shows reduced fold.

Protein name	LB+TMAO	LB+TMAO+150 DIP (Fold changes)
INH21	1	↓ 0.411
FRDA	1	↓ 0.120
PHSM	1	↓ 0.902
KHSE	1	↓ 0.476
AK1H	1	↓ 0.322
ASPG2	1	↓ 0.098
DCDA	1	↓ 0.686
AROH	1	↓ 0.770
THRC	1	↓ 0.420
CARB	1	↓ 0.496
RS6	1	↓ 0.907
TOLC	1	↓ 0.843
PHOE	1	↓ 0.082
MCP1	1	↓ 0.289
LAMB	1	↓ 0.871
DNAA	1	↓ 0.797
MALM	1	↓ 0.842
FLIC	1	↓ 0.156
EX1	1	↓ 0.917
FUMC	1	↓ 0.902
PNP	1	↓ 0.947
PTRA	1	↓ 0.832
PTHA	1	↓ 0.585
SRLD	1	↓ 0.987
T1SK	1	↓ 0.775
BGLR	1	↓ 0.506
TSAD	1	↓ 0.871
BTUB	1	↓ 0.797
CDH	1	↓ 0.709
AGAL	1	↓ 0.609
HEM3	1	↓ 0.988
HISX	1	↓ 0.847
MALT	1	↓ 0.352
PNTA	1	↓ 0.711
PROA	1	↓ 0.930
SDHB	1	↓ 0.444
MCP4	1	↓ 0.042
TYRA	1	↓ 0.662
USHA	1	↓ 0.863
SYV	1	↓ 0.886
CHEA	1	↓ 0.247
RECC	1	↓ 0.874
FDHF	1	↓ 0.164
GLGB	1	↓ 0.612
ILVB	1	↓ 0.622
PUR5	1	↓ 0.825
GLPT	1	↓ 0.600
IDH	1	↓ 0.562
CPDB	1	↓ 0.461
SPPA	1	↓ 0.934
DACC	1	↓ 0.747
T1RK	1	↓ 0.861

T1MK	1	⇩	0.712
MASY	1	⇩	0.458
HEMX	1	⇩	0.767
GALE	1	⇩	0.776
GAL7	1	⇩	0.679
SPEE	1	⇩	0.955
OMPT	1	⇩	0.873
PTW3C	1	⇩	0.563
GLPQ	1	⇩	0.606
PUTA	1	⇩	0.685
GLTB	1	⇩	0.167
GLTD	1	⇩	0.346
REP	1	⇩	0.673
ALR1	1	⇩	0.967
ARGR	1	⇩	0.877
AROK	1	⇩	0.836
ATPE	1	⇩	0.719
BIOD2	1	⇩	0.689
CARA	1	⇩	0.620
GLPK	1	⇩	0.799
CLPX	1	⇩	0.942
CLSA	1	⇩	0.999
KCY	1	⇩	0.896
DADA	1	⇩	0.670
DDLA	1	⇩	0.949
DAPF	1	⇩	0.886
DEF	1	⇩	0.971
NANA	1	⇩	0.798
UXAB	1	⇩	0.181
EFG	1	⇩	0.968
EFP	1	⇩	0.910
FABA	1	⇩	0.859
FIS	1	⇩	0.763
FLGH	1	⇩	0.186
GPDA	1	⇩	0.963
GAL1	1	⇩	0.549
GCSH	1	⇩	0.255
MNMG	1	⇩	0.946
GLGA	1	⇩	0.934
GLGC	1	⇩	0.694
HFQ	1	⇩	0.815
IF2	1	⇩	0.924
IF3	1	⇩	0.797
KTHY	1	⇩	0.930
LPXA	1	⇩	0.918
MGSA	1	⇩	0.801
MSRB	1	⇩	0.296
MURA	1	⇩	0.862
NANE	1	⇩	0.759
NDK	1	⇩	0.917
PYRB	1	⇩	0.918
GPMB	1	⇩	0.870
HSLV	1	⇩	0.965
LEXA	1	⇩	0.768
PEPE	1	⇩	0.280
PTH	1	⇩	0.921
PUR7	1	⇩	0.854

PYRD	1	↓	0.670
SPED	1	↓	0.990
RF1	1	↓	0.724
RF3	1	↓	0.907
RL27	1	↓	0.823
RL32	1	↓	0.940
RL33	1	↓	0.890
RL9	1	↓	0.943
RS11	1	↓	0.983
RS12	1	↓	0.943
RS18	1	↓	0.971
RS20	1	↓	0.876
RS3	1	↓	0.998
RS4	1	↓	1.000
RIMM	1	↓	0.857
RNPA	1	↓	0.888
RPOZ	1	↓	0.948
RUVB	1	↓	0.813
GLYA	1	↓	0.828
SUCC	1	↓	0.841
SURE	1	↓	0.849
TGT	1	↓	0.982
TIG	1	↓	0.933
TNAA	1	↓	0.338
TRPA	1	↓	0.527
TRPB	1	↓	0.509
TYSY	1	↓	0.910
UBIE	1	↓	0.714
YEBC	1	↓	0.979
YEEN	1	↓	0.900
RIMP	1	↓	0.875
YFCN	1	↓	0.986
NRDR	1	↓	0.997
YFBV	1	↓	0.529
YCFP	1	↓	0.685
UPP	1	↓	0.908
UVRC	1	↓	0.547
UXAC	1	↓	0.351
NQOR	1	↓	0.961
TRMB	1	↓	0.915
SYS	1	↓	0.797
SYN	1	↓	0.987
SYT	1	↓	0.491
MATP	1	↓	0.925
EPMA	1	↓	0.895
FRDC	1	↓	0.130
SYDP	1	↓	0.924
METJ	1	↓	0.803
FADR	1	↓	0.989
SLYA	1	↓	0.947
YIHX	1	↓	0.634
BAMC	1	↓	0.989
MIPA	1	↓	0.681
PAL	1	↓	0.868
OMPW	1	↓	0.988
PA1	1	↓	0.810
TSX	1	↓	0.439

MLTA	1	↓	0.886
BAMA	1	↓	0.797
ATDA	1	↓	0.857
FABB	1	↓	0.997
CSPD	1	↓	0.197
CSPE	1	↓	0.791
DPO3B	1	↓	0.857
F16PA	1	↓	0.665
GLPC	1	↓	0.026
FTNA	1	↓	0.112
FTSZ	1	↓	0.870
GALM	1	↓	0.774
GLN1B	1	↓	0.649
GLPX	1	↓	0.739
ACEA	1	↓	0.918
MUG	1	↓	0.737
CHEZ	1	↓	0.178
RBSK	1	↓	0.885
P5CR	1	↓	0.867
PFLA	1	↓	0.707
DEAD	1	↓	0.442
FER	1	↓	0.763
FUCO	1	↓	0.479
GLDA	1	↓	0.762
ETTA	1	↓	0.831
YRDA	1	↓	0.977
CSPC	1	↓	0.748
OMPR	1	↓	0.951
PLAP	1	↓	0.529
QMCA	1	↓	0.982
UBID	1	↓	0.832
SDAC	1	↓	0.333
TDCC	1	↓	0.395
MGLA	1	↓	0.273
PSTB	1	↓	0.914
SAPD	1	↓	0.923
FABF	1	↓	0.910
FABD	1	↓	0.971
HYPB	1	↓	0.122
YBDG	1	↓	0.947
IOJAP	1	↓	0.711
PXPB	1	↓	0.684
YCBJ	1	↓	0.282
YCCJ	1	↓	0.988
LPOB	1	↓	0.851
YCHN	1	↓	0.801
PNTB	1	↓	0.719
KBL	1	↓	0.800
ATP6	1	↓	0.735
ATPF	1	↓	0.766
ATPD	1	↓	0.827
ATPG	1	↓	0.789
ATPA	1	↓	0.842
ATPB	1	↓	0.844
HFLC	1	↓	0.975
BFR	1	↓	0.399
ACCA	1	↓	0.899

FTSI	1	↓	0.950
NLPD	1	↓	0.994
YAJG	1	↓	0.716
LPTG	1	↓	0.845
ILVN	1	↓	0.725
YIFL	1	↓	0.988
YIGB	1	↓	0.563
YIID	1	↓	0.757
RLMM	1	↓	0.991
PPNN	1	↓	0.983
YGIC	1	↓	0.702
LPTA	1	↓	0.968
PPID	1	↓	0.950
RL16	1	↓	0.973
RL23	1	↓	0.869
YAJC	1	↓	0.872
ACRE	1	↓	0.978
AMN	1	↓	0.866
APHA	1	↓	0.415
CHEY	1	↓	0.184
CORC	1	↓	0.788
CPXA	1	↓	0.951
CREA	1	↓	0.979
YNAI	1	↓	0.857
YOAE	1	↓	0.925
USPA	1	↓	0.936
UVRY	1	↓	0.666
DAPE	1	↓	0.805
DGAL	1	↓	0.192
DNAC	1	↓	0.845
DSBA	1	↓	0.960
ELAA	1	↓	0.970
MIAB	1	↓	0.066
RIMO	1	↓	0.694
NUDJ	1	↓	0.804
FABG	1	↓	0.973
FLGM	1	↓	0.218
FLIA	1	↓	0.053
FLIY	1	↓	0.769
FRE	1	↓	0.926
FUCM	1	↓	0.759
GALU	1	↓	0.882
GLNH	1	↓	0.959
GLPF	1	↓	0.786
GUDX	1	↓	0.886
HISJ	1	↓	0.984
HYCA	1	↓	0.803
HYCI	1	↓	0.942
INGK	1	↓	0.733
MALE	1	↓	0.886
MIND	1	↓	0.938
APBC	1	↓	0.604
GHOS	1	↓	0.508
NSRR	1	↓	0.623
YJEI	1	↓	0.797
RIDA	1	↓	0.731
TABA	1	↓	0.978

BOLA	1	⇩	0.805
PCNB	1	⇩	0.827
RODA	1	⇩	0.972
FTSA	1	⇩	0.892
CISY	1	⇩	0.633
CLPA	1	⇩	0.500
DCUA	1	⇩	0.159
DCUC	1	⇩	0.378
GARR	1	⇩	0.661
DKSA	1	⇩	0.808
MENB	1	⇩	0.844
YCHF	1	⇩	0.925
ELBB	1	⇩	0.824
FLIG	1	⇩	0.212
BAMD	1	⇩	0.984
FOLB	1	⇩	0.892
FUMA	1	⇩	0.134
ASPA	1	⇩	0.423
SDHA	1	⇩	0.456
FRDB	1	⇩	0.051
ZUR	1	⇩	0.768
GLO2	1	⇩	0.825
PHSG	1	⇩	0.986
GNSA	1	⇩	0.873
DNAB	1	⇩	0.989
HEMG	1	⇩	0.823
HEMY	1	⇩	0.718
ERPA	1	⇩	0.472
GLMU	1	⇩	0.939
MBHL	1	⇩	0.268
MBHM	1	⇩	0.039
HINT	1	⇩	0.920
DBHA	1	⇩	0.894
DBHB	1	⇩	0.540
HNS	1	⇩	0.840
STPA	1	⇩	0.908
CRP	1	⇩	0.790
DEOR	1	⇩	0.640
FUCR	1	⇩	0.461
GLPR	1	⇩	0.988
EXUR	1	⇩	0.814
CYTR	1	⇩	0.754
CRA	1	⇩	0.382
OXYR	1	⇩	0.936
TDCA	1	⇩	0.512
YEIE	1	⇩	0.774
MPRA	1	⇩	0.780
RPIR	1	⇩	0.667
CECR	1	⇩	0.985
LAPA	1	⇩	0.817
YDCH	1	⇩	0.780
YDJA	1	⇩	0.848
EVGA	1	⇩	0.760
DCUR	1	⇩	0.757
YECF	1	⇩	0.820
YEJL	1	⇩	0.706
YFIA	1	⇩	0.619

NHAB	1	↓	0.705
NTRC	1	↓	0.877
NUOA	1	↓	0.553
NUOB	1	↓	0.303
NUOE	1	↓	0.300
NUOH	1	↓	0.542
NUOI	1	↓	0.233
NUPG	1	↓	0.588
NUSA	1	↓	0.948
ODO1	1	↓	0.708
ODO2	1	↓	0.851
OPPB	1	↓	0.790
OPPC	1	↓	0.802
YJDM	1	↓	0.598
PITA	1	↓	0.857
POTD	1	↓	0.906
PROX	1	↓	0.824
PSPA	1	↓	0.978
GCH1L	1	↓	0.954
BTSR	1	↓	0.578
RFAH	1	↓	0.860
RNK	1	↓	0.278
RSD	1	↓	0.585
SSPB	1	↓	0.941
WZZE	1	↓	0.878
RPE	1	↓	0.822
PURE	1	↓	0.564
SPOT	1	↓	0.930
YIBN	1	↓	0.843
RHO	1	↓	0.999
RL17	1	↓	0.994
RL21	1	↓	1.000
RL30	1	↓	0.913
RS14	1	↓	0.899
RS1	1	↓	0.966
SECB	1	↓	0.791
SECD	1	↓	0.926
SECF	1	↓	0.939
SECE	1	↓	0.955
RPOE	1	↓	0.853
SODF	1	↓	0.141
SRP54	1	↓	0.985
SSB	1	↓	0.984
SUCD	1	↓	0.899
TDCB	1	↓	0.360
TRKA	1	↓	0.943
TDCF	1	↓	0.336
RSME	1	↓	0.896
RL18	1	↓	0.966
IBPA	1	↓	0.723
RELB	1	↓	0.786
SLMA	1	↓	0.814
SRKA	1	↓	0.966
RLME	1	↓	0.884
GATY	1	↓	0.699
KBAZ	1	↓	0.622
EMTA	1	↓	0.801

EFTU1	1	↓	0.963
RCSB	1	↓	0.910
FTSY	1	↓	0.841
GLNQ	1	↓	0.714
FADL	1	↓	0.926
SECA	1	↓	0.970
LPXB	1	↓	0.703
NARV	1	↓	0.327
DAMX	1	↓	0.945
MURF	1	↓	0.897
BIOH	1	↓	0.976
METH	1	↓	0.765
FDHE	1	↓	0.795
KATG	1	↓	0.193
RPOS	1	↓	0.596
TREA	1	↓	0.850
SELB	1	↓	0.971
TOP3	1	↓	0.683
FUMB	1	↓	0.048
AMPP	1	↓	0.975
GLGX	1	↓	0.925
FLIN	1	↓	0.102
SRLR	1	↓	0.563
PUR4	1	↓	0.594
PEPD	1	↓	0.934
PUR9	1	↓	0.763
PUR2	1	↓	0.896
MALQ	1	↓	0.720
SDHL	1	↓	0.573
HYCE	1	↓	0.123
GUTQ	1	↓	0.803
GLMS	1	↓	0.568
UBIG	1	↓	0.929
PNCB	1	↓	0.507
YJJA	1	↓	0.779
AGP	1	↓	0.789
PEPQ	1	↓	0.984
CATE	1	↓	0.809
YCIC	1	↓	0.903
RNE	1	↓	0.915
KPYK2	1	↓	0.807
SYC	1	↓	0.912
RECJ	1	↓	0.775
PCKA	1	↓	0.634
MUKB	1	↓	0.822
MUKE	1	↓	0.837
RIHC	1	↓	0.363
ISPA	1	↓	0.832
TRMA	1	↓	0.963
YIGA	1	↓	0.807
KITH	1	↓	0.879
MUTL	1	↓	0.885
GARL	1	↓	0.654
PPSA	1	↓	0.218
PSS	1	↓	0.990
PHOP	1	↓	0.846
OPPA	1	↓	0.732

DPPA	1	↓	0.725
PSPE	1	↓	0.638
PRC	1	↓	0.990
HEMH	1	↓	0.684
HTPX	1	↓	0.812
MAK	1	↓	0.793
HYPD	1	↓	0.231
HYPE	1	↓	0.296
MRR	1	↓	0.807
YJIA	1	↓	0.513
LPXM	1	↓	0.940
UXUA	1	↓	0.309
CRL	1	↓	0.667
RADA	1	↓	0.963
FRMA	1	↓	0.866
ACNA	1	↓	0.788
HFLX	1	↓	0.997
CODA	1	↓	0.755
ALDA	1	↓	0.786
YIDC	1	↓	0.913
PDXI	1	↓	0.941
HPPK	1	↓	0.736
UBIC	1	↓	0.867
ACUI	1	↓	0.773
PLSC	1	↓	0.759
FTSP	1	↓	0.913
RFAI	1	↓	0.822
RFAJ	1	↓	0.702
WAAU	1	↓	0.814
GCST	1	↓	0.336
NADR	1	↓	0.914
TKT1	1	↓	0.947
EMRA	1	↓	0.695
STHA	1	↓	0.538
RODZ	1	↓	0.960
WECB	1	↓	0.940
WECC	1	↓	0.631
RMLB2	1	↓	0.498
WECE	1	↓	0.946
DCD	1	↓	0.910
ASMA	1	↓	0.926
NRDD	1	↓	0.702
TREC	1	↓	0.253
MENC	1	↓	0.533
PEPT	1	↓	0.622
HCXB	1	↓	0.974
SDHM	1	↓	0.054
BASR	1	↓	0.825
GLMM	1	↓	0.857
ADEC	1	↓	0.289
DGOR	1	↓	0.671
RAVA	1	↓	0.651
YIEP	1	↓	0.777
THIB	1	↓	0.771
LPTD	1	↓	0.877
NUOF	1	↓	0.403
HEMN	1	↓	0.733

YJHC	1	↓	0.787
IADA	1	↓	0.562
BTST	1	↓	0.838
OPGB	1	↓	0.646
GARD	1	↓	0.242
YBAL	1	↓	0.761
OPGD	1	↓	0.851
PDXK	1	↓	0.939
NANT	1	↓	0.318
RIHA	1	↓	0.590
RLMG	1	↓	0.841
YGJR	1	↓	0.767
TDCG	1	↓	0.170
TDCE	1	↓	0.271
HRPA	1	↓	0.976
YHCH	1	↓	0.843
NANK	1	↓	0.681
PROQ	1	↓	0.885
HEXR	1	↓	0.486
YHGF	1	↓	0.911
MLC	1	↓	0.768
YAIL	1	↓	0.870
SFGH1	1	↓	0.674
IXTPA	1	↓	0.896
RSTA	1	↓	0.835
RATB	1	↓	0.944
NIFJ	1	↓	0.660
PTHC	1	↓	0.912
PTHB	1	↓	0.605
MUKF	1	↓	0.951
TRUB	1	↓	0.865
RSMH	1	↓	0.986
RL3	1	↓	0.970
GUAC	1	↓	0.920
RL24	1	↓	0.955
HIS6	1	↓	0.824
LIPA	1	↓	0.283
RL4	1	↓	0.934
LEPA	1	↓	0.988
YIDE	1	↓	0.938
LGT	1	↓	0.895
MDH	1	↓	0.895
ISPF	1	↓	0.993
ISPH	1	↓	0.903
RLMB	1	↓	0.767
GADE	1	↓	0.888
MLAF	1	↓	0.923
YHES	1	↓	0.834
YHBS	1	↓	0.703
AMIC	1	↓	0.765
ZNTB	1	↓	0.973
CNU	1	↓	0.934
YOAC	1	↓	0.603
YFCL	1	↓	0.777
QUEE	1	↓	0.999
SDHE	1	↓	0.555
YGGT	1	↓	0.693

CSQR	1	↓	0.622
FDOG	1	↓	0.065
SBMC	1	↓	0.868
SFGH2	1	↓	0.763
GCSP	1	↓	0.520
TORA	1	↓	0.118
TORC	1	↓	0.070
YEHZ	1	↓	0.899
BGLX	1	↓	0.711
NIKA	1	↓	0.897
NIKD	1	↓	0.743
NIKE	1	↓	0.858
NUOCD	1	↓	0.551
NUOG	1	↓	0.233
YEJF	1	↓	0.797
NAPA	1	↓	0.157
AMIA	1	↓	0.892
DSBD	1	↓	0.774
PTTBC	1	↓	0.205
ACNB	1	↓	0.372
RSMB	1	↓	0.909
PGM	1	↓	0.983
FXSA	1	↓	0.751
HYBD	1	↓	0.171
PTKB	1	↓	0.384
MASZ	1	↓	0.926
LHGD	1	↓	0.909
YJJI	1	↓	0.668
PSPF	1	↓	0.691
UCPA	1	↓	0.424
PMRD	1	↓	0.294
YHII	1	↓	0.713
GADX	1	↓	0.969
KDGK	1	↓	0.808
ALDB	1	↓	0.970
RFAF	1	↓	0.899
RMLA1	1	↓	0.849
RMLC	1	↓	0.905
WBBI	1	↓	0.859
WBBK	1	↓	0.758
RMLB1	1	↓	0.644
RMLD	1	↓	0.708
RLUB	1	↓	0.731
GLTI	1	↓	0.987
USPF	1	↓	0.689
MENF	1	↓	0.958
YGGL	1	↓	0.855
DEGQ	1	↓	0.917
UXUB	1	↓	0.262
ZNUA	1	↓	0.920
USPG	1	↓	0.836
AG43	1	↓	0.937
RSGA	1	↓	0.996
YTFB	1	↓	0.968
QOR2	1	↓	0.933
TAMB	1	↓	0.862
YTFQ	1	↓	0.431

YQJI	1	↓	0.937
YRAP	1	↓	0.987
YHBT	1	↓	0.234
MLAE	1	↓	0.923
YFCD	1	↓	0.993
QUED	1	↓	0.881
DIAA	1	↓	0.907
RSMI	1	↓	0.974
YQFB	1	↓	0.749
YHAJ	1	↓	0.882
MALG	1	↓	0.908
RS21	1	↓	0.941
ATPL	1	↓	0.794
AMPA	1	↓	0.951
DHSC	1	↓	0.616
IF1	1	↓	0.915
TATB	1	↓	0.756
TATA	1	↓	0.823
KAD	1	↓	0.723
MBHS	1	↓	0.052
MBHT	1	↓	0.005
PTGCB	1	↓	0.938
PTQB	1	↓	0.649
PTNAB	1	↓	0.814
PTKA	1	↓	0.542
PTSN	1	↓	0.994
PTKC	1	↓	0.534
DMSD	1	↓	0.264
POTA	1	↓	0.903
FCTA	1	↓	0.951
FUCI	1	↓	0.488
WCAJ	1	↓	0.322
YAGE	1	↓	0.258
YBFF	1	↓	0.947
PXPC	1	↓	0.821
PXPA	1	↓	0.704
RLMF	1	↓	0.730
YBJS	1	↓	0.869
LTAE	1	↓	0.980
HCR	1	↓	0.117
HCP	1	↓	0.097
YBJX	1	↓	0.381
YCBX	1	↓	0.627
LONH	1	↓	0.844
YCDX	1	↓	0.875
YCDY	1	↓	0.889
NAGZ	1	↓	0.729
NPD	1	↓	0.737
YCGM	1	↓	0.854
DHAL	1	↓	0.909
DHAK	1	↓	0.830
OPPD	1	↓	0.529
YCIT	1	↓	0.931
TTCA	1	↓	0.762
RACR	1	↓	0.991
YDCS	1	↓	0.777
DMLA	1	↓	0.974

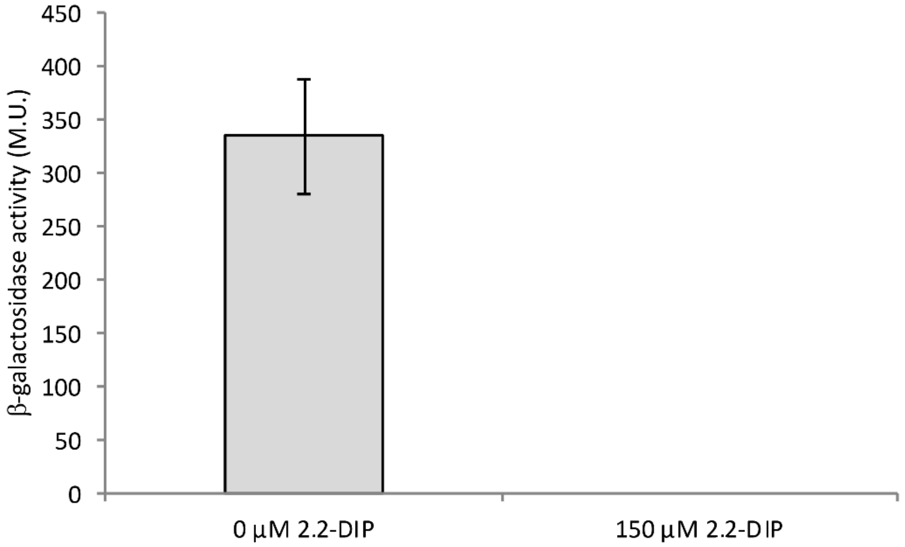

Figure S1: β-galactosidase activity in Miller units was determined for *torC-lacZ* fusion in *E. coli* BW25113 strain in the presence or absence of 150 µM 2.2 DIP to confirm the regulation by iron availability as observed in the proteomic data. The strains were cultivated anaerobically in the presence of 20 mM TMAO for 4 h. β-galactosidase activities were normalized to the $OD_{600\ nm}$ values. Standard deviation was calculated from three biological replicates.

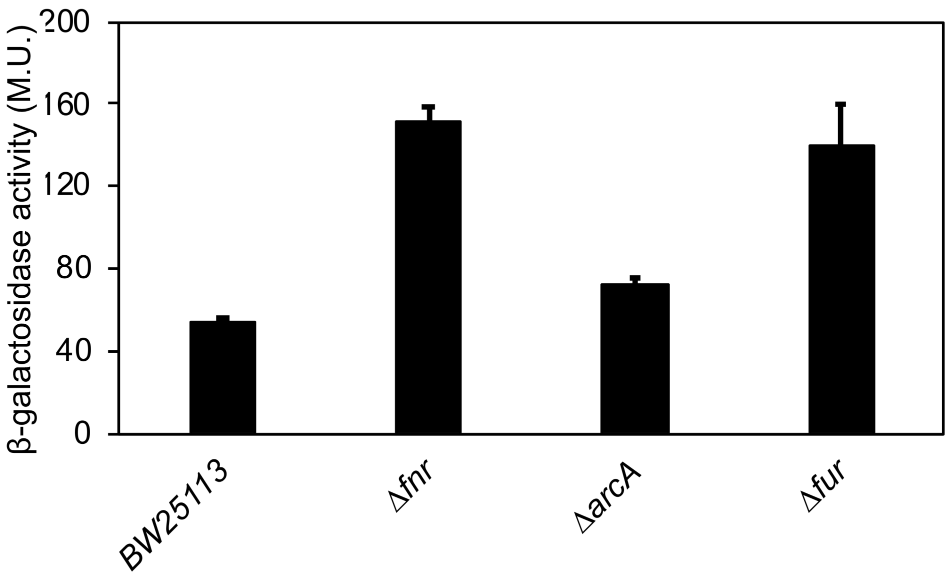

Figure S2: **Expression of a *fnr-lacZ* fusion in different mutant strains.**

β-galactosidase activity in the Miller unit was determined for *fnr-lacZ* fusion in *E. coli* mutant strains to reveal the regulation by FNR, Fur and ArcA. The strains were cultivated anaerobically in the presence of 20 mM TMAO for 4 h. β-galactosidase activities were normalized to the $OD_{600\ nm}$ values. Standard deviation was calculated from three biological replicates.

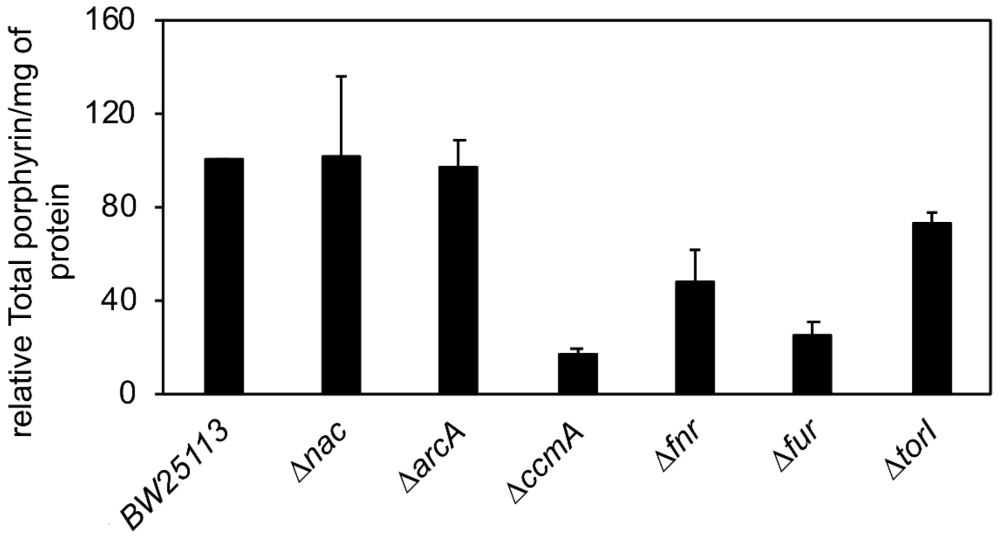

Figure S3: **Quantification of total porphyrin in different *E. coli* strains.**

Total porphyrin content determination in the *E. coli* BW25113 wildtype strain and the mutant Δ*nac,* Δ*arcA,* Δ*ccmA,* Δ*fnr,* Δ*fur* and *ΔtorI* strains. The strains were grown at 37 °C under anaerobic conditions using 20 mM TMAO. Total porphyrin fluorescence intensity in the strains was determined by an excitation of 409 nm and an emission of 630 nm. Fluorescence intensity was normalized to the protein concentration and the levels of wildtype BW25113 strain was set to 100%.

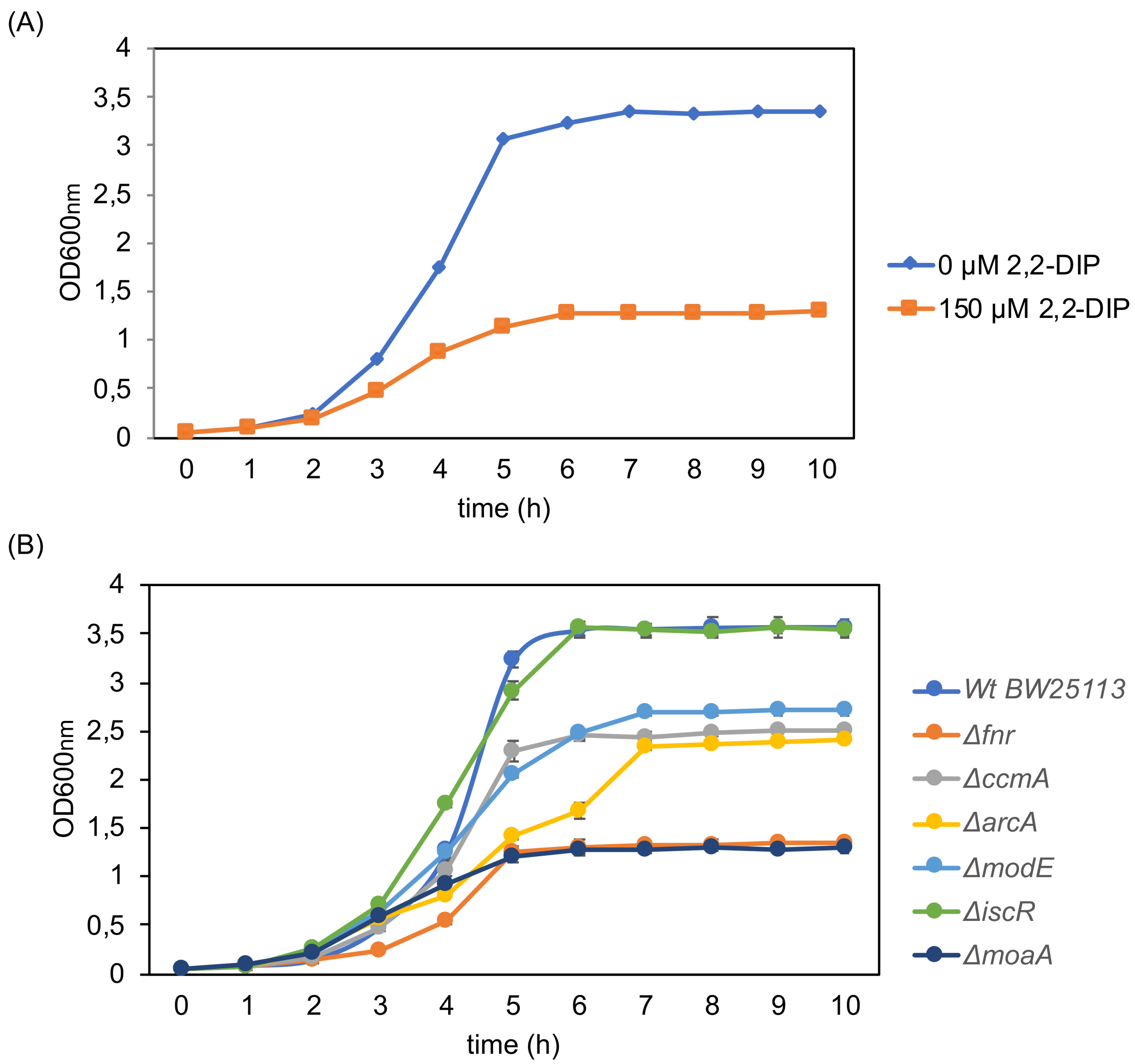

Figure S4. Growth curves of BW25113 parental strain in dependence of (A) dipyridyl and (B) different mutant strains. (A) Growth curves of strains BW25113 (blue) and BW25113 in presence of 150 µM dipyridyl (red) in LB medium anaerobic conditions. (B) growth curves of strains Δ*fnr* (red), Δ*ccmA* (grey), Δ*arcA* (yellow), Δ*modE* (light blue), Δ*iscR* (green) Δ*moaA* (dark blue). Standard deviation was calculated from three biological replicates.

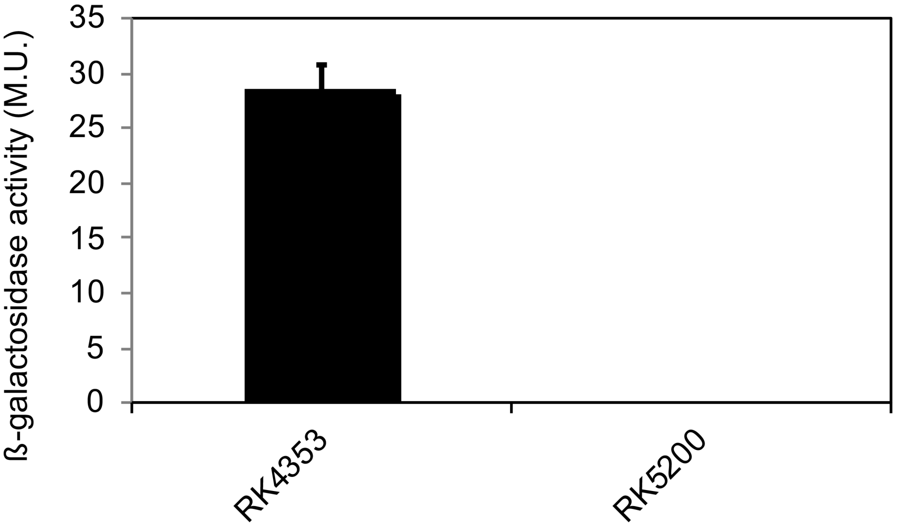

Figure S5: **Expression of a *torC-lacZ* fusion in different RK4353 strains.**

β-galactosidase activity in the Miller unit was determined for *torC-lacZ* (pPTor3) fusion in *E. coli* RK4353 and RK5200 (Δ*moaA*) strains. The strains were cultivated anaerobically in the presence of 20 mM TMAO for 4 h. β-galactosidase activities were normalized to the $OD_{600\ nm}$ values. Standard deviation was calculated from three biological replicates.

References:

1. Jourlin, C., Simon, G., Lepelletier, M., Chippaux, M., and Mejean, V. (1995) Conservation of cis-acting elements within the tor regulatory region among different Enterobacteriaceae. *Gene* **152**, 53-57
2. Ansaldi, M., Bordi, C., Lepelletier, M., and Mejean, V. (1999) TorC apocytochrome negatively autoregulates the trimethylamine N-oxide (TMAO) reductase operon in Escherichia coli. *Mol Microbiol* **33**, 284-295
3. Buhning, M., Valleriani, A., and Leimkuhler, S. (2017) The Role of SufS Is Restricted to Fe-S Cluster Biosynthesis in Escherichia coli. *Biochemistry* **56**, 1987-2000
4. Park, D. M., Akhtar, M. S., Ansari, A. Z., Landick, R., and Kiley, P. J. (2013) The bacterial response regulator ArcA uses a diverse binding site architecture to regulate carbon oxidation globally. *PLoS Genet* **9**, e1003839
5. Tran, Q. H., Arras, T., Becker, S., Holighaus, G., Ohlberger, G., and Unden, G. (2000) Role of glutathione in the formation of the active form of the oxygen sensor FNR ([4Fe-4S].FNR) and in the control of FNR function. *Eur J Biochem* **267**, 4817-4824
6. Simon, G., Mejean, V., Jourlin, C., Chippaux, M., and Pascal, M. C. (1994) The torR gene of Escherichia coli encodes a response regulator protein involved in the expression of the trimethylamine N-oxide reductase genes. *J Bacteriol* **176**, 5601-5606
7. Hänzelmann, P., Hernandez, H. L., Menzel, C., Garcia-Serres, R., Huynh, B. H., Johnson, M. K., Mendel, R. R., and Schindelin, H. (2004) Characterization of MOCS1A, an oxygen-

sensitive iron-sulfur protein involved in human molybdenum cofactor biosynthesis. *J Biol Chem* **279**, 34721-34732
8. Baba, T., Ara, T., Hasegawa, M., Takai, Y., Okumura, Y., Baba, M., Datsenko, K. A., Tomita, M., Wanner, B. L., and Mori, H. (2006) Construction of Escherichia coli K-12 in-frame, single-gene knockout mutants: the Keio collection. *Molecular systems biology* **2**, 2006 0008
9. Stewart, V., and MacGregor, C. H. (1982) Nitrate reductase in *Escherichia coli* K-12: involvement of *chlC*, *chlE*, and *chlG* loci. *J. Bacteriol.* **151**, 788-799
